# A systematic review of cognitive behavioral therapy-based interventions for comorbid chronic pain and clinically relevant psychological distress

**DOI:** 10.3389/fpsyg.2023.1200685

**Published:** 2023-12-22

**Authors:** Juan P. Sanabria-Mazo, Ariadna Colomer-Carbonell, Óscar Fernández-Vázquez, Georgina Noboa-Rocamora, Gemma Cardona-Ros, Lance M. McCracken, Antonio Montes-Pérez, Juan R. Castaño-Asins, Sílvia Edo, Xavier Borràs, Antoni Sanz, Albert Feliu-Soler, Juan V. Luciano

**Affiliations:** ^1^Teaching, Research, and Innovation Unit, Parc Sanitari Sant Joan de Déu, Sant Boi de Llobregat, Spain; ^2^Department of Basic, Developmental and Educational Psychology, Autonomous University of Barcelona, Barcelona, Spain; ^3^Centre for Biomedical Research in Epidemiology and Public Health (CIBERESP), Madrid, Spain; ^4^Faculty of Psychology, University of Barcelona, Barcelona, Spain; ^5^Psychology Department, Uppsala University, Uppsala, Sweden; ^6^Parc de Salut MAR, Barcelona, Spain; ^7^Department of Clinical and Health Psychology, Autonomous University of Barcelona, Barcelona, Spain

**Keywords:** cognitive behavioral therapy, chronic pain, distress, depression, anxiety, systematic review

## Abstract

**Objective:**

Chronic pain frequently co-occurs with clinically relevant psychological distress. A systematic review was conducted to identify the efficacy of cognitive behavioral therapy-based interventions for patients with these comorbid conditions.

**Methods:**

The systematic search was carried out in Medline, PsycINFO, Web of Science, and Scopus up to March 18th, 2023. Four reviewers independently conducted screenings, extraction, and quality assessment.

**Results:**

Twelve randomized controlled trials and one non-randomized controlled trial involving 1,661 participants that examined the efficacy of Cognitive Behavioral Therapy (nine studies), Mindfulness-based Interventions (three studies), Acceptance and Commitment Therapy (one study), and Behavioral Activation Therapy for Depression (one study) were included. Compared to treatment as usual, six out of eight studies of traditional Cognitive Behavioral Therapy reported significant differences in the reduction of depressive symptoms at post-treatment (*d* from 1.31 to 0.18) and four out of six at follow-up (*d* from 0.75 to 0.26); similarly, five out of six reported significant differences in the reduction of anxiety symptoms at post-treatment (*d* from 1.08 to 0.19) and three out of four at follow-up (*d* from 1.07 to 0.27). Overall, no significant differences between traditional Cognitive Behavioral Therapy and treatment as usual were reported at post-treatment and follow-up in the studies exploring pain intensity and pain catastrophizing.

**Conclusion:**

The available evidence suggests that traditional Cognitive Behavioral Therapy may produce significant benefits for the improvement of depression, anxiety, and quality of life, but not for pain intensity and pain catastrophizing. More evidence is needed to determine the effects of MBI, ACT, and BATD.

**Systematic review registration:**

PROSPERO, CRD42021219921.

## 1 Introduction

Chronic pain and psychological distress are common health conditions (Wittchen et al., 2011) with substantial healthcare and social impacts (Chopra and Arora, 2014). The prevalence of chronic pain ranges from 10% to 30% worldwide (Reid et al., 2011), generating a significant public health demand and economic burden (Baumeister et al., 2012). According to epidemiological studies, comorbidity between chronic pain and psychological distress in clinical practice is higher than 60% (Walker et al., 2014). Since this comorbidity is more treatment-resistant than either condition alone (Mansfield et al., 2016) and it generates a significant impact on the quality of life of patients with these conditions (McCracken et al., 2022), it has been considered a growing target for treatment in recent years (McCracken, 2023). The concurrent appearance of chronic pain and significant psychological distress is striking and requires attention from researchers, clinicians, and policymakers, as well as demands effective management strategies to improve the health and well-being of those affected by these conditions (Snyder and Handrup, 2018).

Due to the complexity and multifaceted nature of the construct, many definitions for psychological distress have been proposed in recent years. One of the most widely accepted defines this psychological construct as “state of emotional suffering characterized by the undifferentiated combinations of symptoms of depression (e.g., lost interest, sadness, hopelessness) and anxiety (e.g., restlessness, feeling tense) which are sometimes accompanied by somatic symptoms (e.g., insomnia, headaches, lack of energy)” (Drapeau et al., 2012, p. 125). Generally, psychological distress refers to a range of unpleasant emotional and mental experiences that can impact a person’s well-being and ability to function (Bisby et al., 2022; Gasslander et al., 2022). It is also considered a dimensional construct that has been truncated in most studies to employ it as a categorical construct to establish when it is or is not “clinically relevant,” with relevant meaning that scores on psychopathological measures exceed specific cut-off points.

Previous studies demonstrate that people with chronic pain are more likely to experience psychological distress, such as anxiety and depression, and individuals with psychological distress are more likely to report chronic pain (Rayner et al., 2016). The relationship between chronic pain and psychological distress is complex and bidirectional (Wittchen et al., 2011). The multidimensional nature of both chronic pain and psychological distress, with sensory, affective, and behavioral dimensions, is a challenge for intervention design and delivery (Roberts et al., 2018). Specifically, the presence of psychological distress in patients with chronic pain increases pain complaints and reduces quality of life (Snyder and Handrup, 2018). Comorbidity between psychological distress and chronic pain generates a higher degree of functional impairment than the presence of either condition alone (Mansfield et al., 2016) and negatively influences the response to pharmacological and non-pharmacological treatments (Kroenke et al., 2011). Chronic pain and clinical psychological distress involve shared neurobiological and psychosocial processes (Hooten, 2016).

Cognitive Behavioral Therapy (CBT) is the most applied psychological approach to chronic pain (McCracken, 2023). Different forms of CBT are frequently applied in chronic pain and related conditions (e.g., anxiety and/or depression), appearing effective when explored independently (Churchill et al., 2013; Cuijpers et al., 2013; Buhrman et al., 2016; Pasarelu et al., 2017). Traditional CBT has beneficial effects in adults with chronic pain (Williams et al., 2020) and is also effective in patients with emotional disorders (Lorenzo-Luaces et al., 2018; López-López et al., 2019). Concretely, recent evidence shows that Mindfulness-based Interventions (MBI), Dialectical Behavior Therapy (DBT), Rational Emotive Behavior Therapy (REBT), Acceptance and Commitment Therapy (ACT), and Behavioral Activation Therapy for Depression (BATD) also produce positive effects in patients with chronic pain (Jorn, 2015; Veehof et al., 2016; Hughes et al., 2017; Boersma et al., 2019; Khoo et al., 2019; Gloster et al., 2020; Pardos-Gascón et al., 2021).

Although the above-mentioned CBT-based interventions have generally demonstrated evidence in the management of chronic pain and related conditions, their specific efficacy in patients with comorbid pain and clinical psychological distress has been scarcely assessed. It appears that this is the first systematic review that aims to examine the efficacy of CBT-based interventions for comorbid chronic pain and clinically relevant psychological distress. Since chronic pain and psychological distress frequently co-occur, worsen one another, and resist therapy effects when they are both present, identifying effective CBT-based interventions for this complex set of conditions is critical work. In this systematic review, randomized controlled trials (RCTs) and non-randomized trials (non-RCTs) were selected for patients with chronic pain plus clinically relevant psychological distress, comparing CBT-based interventions to control conditions (active or inactive). Additionally, this research explored the risk of bias (RoB) of the included studies to assess their methodological quality.

## 2 Methods

### 2.1 Protocol and registration

This systematic review was performed following the Preferred Reporting Items for Systematic Reviews and Meta-Analyses statement (PRISMA; Page et al., 2021). The review protocol was registered in the Prospective Register of Systematic Reviews (PROSPERO), under identification number CRD42021219921. Supplementary Table S1 indicates some adjustments incorporated into the protocol of this systematic review and includes the PRISMA checklist.

### 2.2 Data sources and searches

To reduce publication bias, published and unpublished clinical trials were examined. For exploration of published clinical trials, searches were conducted in four electronic databases: Medline (PubMed), Web of Science (Core Collection), PsycINFO (ProQuest), and Scopus (Elsevier). The search strategy identified studies that included combinations of the population terms and the specific terms of psychological therapies. The search terms were selected according to a validation by experts and a review of the search strategies used in previous systematic reviews on CBT-based interventions for chronic pain (Lin et al., 2019; Williams et al., 2020; White et al., 2022). The specific Boolean searches were adjusted according to the Peer Review of Electronic Search Strategies (PRESS) guideline statement (McGowan et al., 2016). The following limits and filters were activated in all databases if possible: publication date (from inception until March 18th, 2023), type of publication (only studies of interest), species (humans), and languages (English and Spanish). The bibliographic database searches are detailed in Table 1.

**Table 1:** Bibliographic database searches.

Databases: Medline, Web of Science, PsycINFO, and Scopus	
1	Chronic pain [Title/Abstract] OR eye pain [Title/Abstract] OR neck pain [Title/Abstract] OR nociceptive pain [Title/Abstract] OR facial pain [Title/Abstract] OR shoulder pain [Title/Abstract] OR myofascial pain syndromes [Title/Abstract] OR pelvic pain [Title/Abstract] OR patellofemoral pain syndrome [Title/Abstract] OR pelvic girdle pain [Title/Abstract] OR abdominal pain [Title/Abstract] OR flank pain [Title/Abstract] OR low back pain [Title/Abstract] OR back pain [Title/Abstract] OR musculoskeletal pain [Title/Abstract] OR chest pain [Title/Abstract] OR complex regional pain syndromes [Title/Abstract] OR visceral pain [Title/Abstract] OR neuropath* [Title/Abstract] OR phantom limb [Title/Abstract] OR fantom limb [Title/Abstract] OR spinal cord [Title/Abstract] OR idiopathic [Title/Abstract] OR shoulder [Title/Abstract] OR persistent sciatica [Title/Abstract] OR lumbago [Title/Abstract] OR fibromyalgia [Title/Abstract] OR complex regional pain syndromes [Title/Abstract] OR headache disorders [Title/Abstract]
2	Depress* [Title/Abstract] OR anxi* [Title/Abstract] OR stress [Title/Abstract] OR distress [Title/Abstract] OR mood disorder [Title/Abstract] OR emotional regulation [Title/Abstract] OR emotional dysregulation [Title/Abstract] OR affective disorder [Title/Abstract]
3	Intervention [Title/Abstract] OR treatment [Title/Abstract] OR psychotherapy [Title/Abstract] OR therapy [Title/Abstract] OR clinical trial [Title/Abstract] OR trial [Title/Abstract] OR cognitive behavioral therapy [Title/Abstract] OR mindfulness [Title/Abstract] OR acceptance and commitment therapy [Title/Abstract] OR behavioral activation therapy [Title/Abstract]
((1 AND 2) AND 3)	

The following filters were applied in all databases if possible: type of publication (controlled trials only), species (humans), and languages (English and Spanish).

For the exploration of unpublished clinical trials, a search was conducted in ClinicalTrials.Gov, International Standard Randomized Controlled Trial Number register (ISRCTN), World Health Organization (WHO) International Clinical Trials Registry Platform (ICTRP), and PROSPERO (Lin et al., 2019). The reference list of included articles was also examined through a reverse citation search for further analysis. In addition, the reference list of published narrative reviews, systematic reviews, and meta-analyses, as well as grey literature (search carried out in Google Scholar), were consulted to ensure that all eligible studies were included (i.e., Buhrman et al., 2016; Hilton et al., 2017; Ahern et al., 2018; Haugmark et al., 2019; Khoo et al., 2019; López-López et al., 2019; Williams et al., 2020; Fordham et al., 2021; Pardos-Gascón et al., 2021; White et al., 2022).

### 2.3 Eligibility criteria

To select the eligibility criteria, the “Population,” “Intervention,” “Comparison,” “Outcomes,” and “Study” (PICOS) approach was followed. Table 2 details the inclusion and exclusion criteria established in this systematic review.

**Table 2:** Eligibility criteria according to PICOS strategy.

	Inclusion criteria	Exclusion criteria
[P] Participants	Adults (≥ 18 years) with the presence of non-oncologic chronic pain (> 12 weeks) and clinically relevant psychological distress	Adults diagnosed with psychiatric disorders other than depression and/or anxiety, other clinically relevant psychiatric symptoms, substance dependence, and neurodegenerative disorders
[I] Intervention	CBT-based interventions exploring their efficacy in patients with non-oncologic chronic pain and clinically relevant psychological distress	The combination of pharmacological and CBT-based interventions
[C] Comparison	CBT-based interventions compared with active (i.e., another type of psychological intervention) or inactive treatment (i.e., wait-list, usual care, attention control, and psychological placebo, among others)	Interventions without a control group
[O] Outcomes	Pain-related variables (pain interference, pain intensity, pain acceptance, pain catastrophizing, and pain self-efficacy, among others), emotional functioning (depression, anxiety, and stress), health-related quality of life, behavioral activation, and psychological flexibility, among others	Other types of outcomes
[S] Study design	RCTs and non-RCTs	Research with other study designs

#### 2.3.1 Participants

The population of interest consisted of adults (≥ 18 years) with the presence of non-oncologic chronic pain (> 12 weeks) and clinically relevant psychological distress, according to the clinical cut-off for depression and/or anxiety reported in the studies. Participants diagnosed with psychiatric disorders other than depression and/or anxiety, other clinically relevant psychiatric symptoms, substance dependence, and neurodegenerative disorders were excluded.

#### 2.3.2 Interventions

CBT-based interventions exploring their efficacy in patients with non-oncologic chronic pain and clinically relevant psychological distress, regardless of their mode of delivery (e.g., face-to-face, online, and blended format). To explore all available evidence in the literature, this systematic review synthesized the efficacy of all CBT-based interventions that met this eligibility criteria. The points analyzed for each outcome were the post-treatment and the follow-up assessment, examining differences between the groups. The combination of pharmacological and CBT-based interventions was excluded.

#### 2.3.3 Comparators

CBT-based interventions were included exclusively when the comparison group received active (i.e., another type of psychological intervention) or inactive treatment (i.e., wait-list, usual care, attention control, and psychological placebo, among others). Given the objective of this study, CBT-based interventions without a control group were excluded.

#### 2.3.4 Outcomes

The selection of outcomes was based on recommendations from the Initiative on Methods, Measurement, and Pain Assessment in Clinical Trials (IMMPACT; Dworkin et al., 2008). Specifically, pain-related variables (pain interference, pain intensity, pain acceptance, pain catastrophizing, and pain self-efficacy, among others), emotional functioning (depression, anxiety, and stress), health-related quality of life, behavioral activation, and psychological flexibility, among others, were explored in this systematic review.

#### 2.3.5 Study design

RCTs and non-RCTs of any length of follow-up were included. Only data from studies that have received ethical approval and were published in English or Spanish were used. No studies were excluded based on publication status, date, or type (Lin et al., 2019).

### 2.4 Data management and study selection

Duplicate articles in the databases were automatically removed by Mendeley. Then, four reviewers independently screened all articles in Rayyan QCRI based on their titles and abstracts. The full texts were independently checked for compliance with the eligibility criteria. Finally, the reviewers entered key information from each study into a standardized data extraction form and assessed the RoB of included studies. During each phase, at least two reviewers were employed. No additional reviewer was needed to resolve a disagreement.

### 2.5 Risk of bias

The RoB of the included studies was assessed using the Cochrane Collaboration’s risk of bias assessment tool (Higgins et al., 2011). This tool involves the assessment of RoB arising from each of six domains: selection bias, performance bias, detection bias, attrition bias, reporting bias, and other biases. Studies were classified as high risk (if at least one domain was assessed as high), unclear (if at least one domain was assessed as unclear and the other domains were low), or low risk of bias (if all individual domains were low).

### 2.6 Data synthesis

Findings were described according to therapy type (CBT, MBI, ACT, and BATD). A narrative synthesis was carried out to describe the main characteristics of psychological therapies and the results obtained in the comparison of outcomes with control conditions (inactive or active). The statistical significance threshold was set at *p* < 0.05 and the magnitude of Cohen’s *d* was interpreted according to the following rule of thumb criterion (Sawilowsky, 2009): very small (0.10), small (0.20), medium (0.50), large (0.80), very large (1.20), and huge (2.00).

## 3 Results

### 3.1 Selection and inclusion of studies

**Figure 1:**
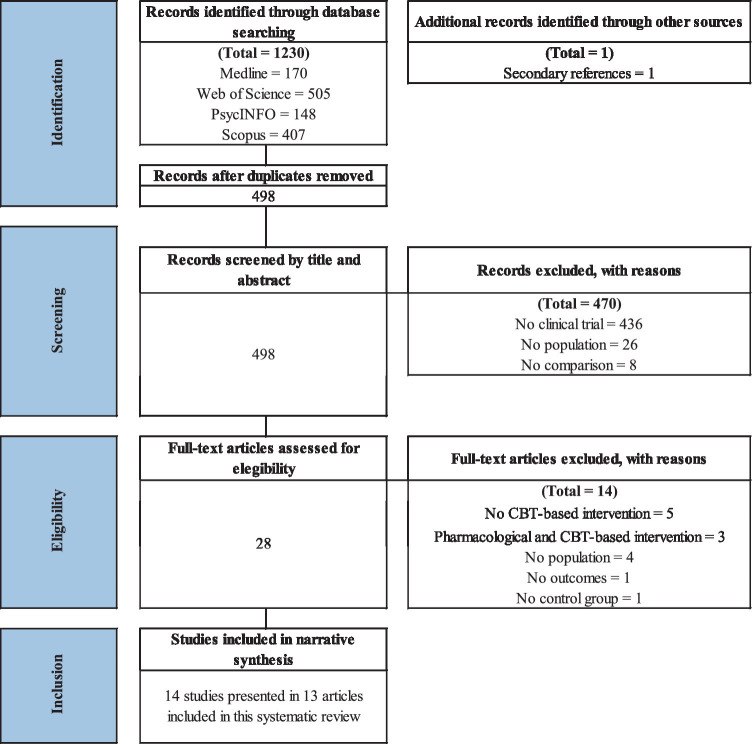
Preferred Reporting Items for Systematic Reviews and Meta-analyses (PRISMA) flowchart from record identification to study inclusion.

The initial database search yielded a total of 1,230 published articles. As shown in Figure 1, after removing duplicates and screenings, 14 articles based on 12 RCT and 1 non-RCT were included. Two studies were derived from the same sample (De Jong et al., 2016, 2018), although they presented evidence of different outcomes. The 14 articles that were excluded during the full-text screening are presented in Supplementary Table S2.

### 3.2 Characteristics of all the included studies

The 13 articles included were published between 2011 and 2023. Three studies (23%) were conducted in Sweden, three (23%) in Spain, three (23%) in Germany, two (15%) in the United States of America, one (8%) in Australia, and one (8%) in Iceland. Five studies (38%) assessed patients with chronic pain (CP), three (23%) with chronic low back pain (CLBP), two (15%) with chronic musculoskeletal pain (CMP), one (8%) with chronic back pain (CBP), one (8%) with chronic spinal cord injury, and one (8%) with non-specific chronic pain (NSCP). Nine studies (69%) included CBT, three (23%) MBI, and one ACT and BATD (8%) as the main therapy of interest. Eleven studies (85%) employed inactive control groups (usual care or waitlist). All the studies (100%) carried out the therapy program weekly. The format of the therapy was face-to-face in six studies (46%), entirely online in five (38%), a blended format in one (8%), and combined face-to-face plus online versus online in one (8%).

The sample size of the study’s arms ranged from 26 to 167 in the intervention group (IG) and 24 to 161 in the control group (CG), and the mean age varied from 45 to 61 in IG and 46 to 59 years old in CG. In total, 1,661 participants were involved in this systematic review, of which 850 were in IG and 811 in CG. The proportion of women in all studies was higher than 50%, both in IG and CG, except for the IG in two (43.2% and 26%; Tlach and Hampel, 2011; Migliorini et al., 2016, respectively) and CG in one (32%; Migliorini et al., 2016). The employment status was reported in nine studies (69.2%) and medication consumption in eight studies (61.6%). The dropout rate at the end of the studies ranged from 17 to 67%. The number of sessions ranged from four to thirteen with a minimum duration of 50 min per session and a maximum of 150 min. The therapies were delivered by psychologists in ten studies (77.7%), other professionals in two (27.3%), and without therapists in one (7.7%). Details are described in Table 3.

**Table 3:** Characteristics of the controlled trials included in the systematic review.

Author (year), country	Target condition (measure), study design	Treatment arms (sample) and delivery period (format)	Groups (intervention and control): age and gender	Components (dropout and adherence rate), sessions (duration), and therapist	Assessments (time horizon)	Primary outcome (instrument)	Secondary outcomes (instruments)
Cognitive behavioral therapy (CBT)							
[1] Tlach and Hampel (2011), Germany	Patients with CLBP and depression (measured with the ADS; cut-off ≥24 points), non-RCT	*Treatment arms:* CBT + TAU (*n* = 44) and TAU (*n* = 40) *Delivery period:* weekly (face-to-face)	*Intervention group*: CBT + TAU. Age: *M* = 50.08 (*SD* = 5.4). Gender: 25 females (43.2%) *Control group:* TAU. Age: *M* = 51.00 (*SD* = 6.3). Gender: 20 females (50.0%)	*Components:* a biopsychosocial approach of CBT: cognitive-behavioral pain-management training and cognitive-behavioral training program for the management of depressive symptoms (37% dropout rate at the end of the study; adherence rate was not reported) *Number of sessions:* 13 (60 min) *Therapist:* physicians and nurses	Pre, post, follow-up+6, follow-up+12, and follow-up+24 (24 months)	Depression symptoms (CES-D)	Anxiety (HADS-A) Mental quality of life (SF-12)
[2] Buhrman et al. (2015), Sweden	Patients with CP and depression (measured with the MADRS-S; cut-off >10 points), RCT	*Treatment arms:* CBT + TAU (*n* = 28) and TAU (*n* = 24) *Delivery period:* weekly (online)	*Intervention group*: CBT + TAU. Age: *M* = 54.1 (*SD* = 11.76). Gender: 24 females (86%) *Control group:* TAU. Age: *M* = 46.8 (*SD* = 12.9). Gender: 20 females (83%)	*Components:* program based on CBT: behavioral activation and psychoeducation (17% dropout rate at the end of the study and 44% completed 100% of the total number of sessions) *Number of sessions:* 8 (NR minutes) *Therapist:* graduate students trained in CBT with supervision by a clinical psychologist	Pre, post, and follow-up+12 (12 months)	Depression symptoms (MADRS-S) Anxiety symptoms (BAI) Pain interference (PDI)	Fear of the symptoms of anxiety (ASI) Pain catastrophizing (PCS) Chronic pain acceptance (CPAQ) Cognitive and behavioral coping strategies (CSQ) Psychosocial and behavioral consequence of chronic pain (MPI) Quality of life (QoLI)
[3] Migliorini et al. (2016), Australia	Patients with chronic spinal cord injury and depression or anxiety (measured with the DASS-21; cut-off ≥ was not reported), RCT	*Treatment arms:* CBT (*n* = 34) and waitlist (*n* = 25) *Delivery period:* weekly (face-to-face)	*Intervention group*: CBT. Age: *M* = 47.5 (*SD* = 12.2). Gender: 9 females (26%) *Control group:* Waitlist. Age: *M* = 52.8 (*SD* = 12.9). Gender: 8 females (32%)	*Components:* internet program based on CBT: psychoeducation, mindfulness, and positive psychology (32% dropout rate at the end of the study; adherence rate was not reported) *Number of sessions:* 10 (NR minutes) *No therapist*s	Pre and post	Depression, anxiety, and stress symptoms (DASS-21)	Quality of life (PWIA)
[4] Ólason et al. (2018), Iceland	Patients with CP and depression or anxiety (measured with the BDI-II or BAI; cut-off ≥ was not reported), RCT	*Treatment arms:* CBT + TAU (*n* = 39) and TAU (*n* = 38) *Delivery period:* weekly (face-to-face)	*Intervention group*: CBT + TAU. Age: *M* = 37.32 (*SD* = 12.16). Gender: 21 females (59%) *Control group:* TAU. Age: *M* = 35.79 (*SD* = 11.28). Gender: 26 females (68%)	*Components:* a biopsychosocial approach of CBT: pain and emotional management training (34% dropout rate at the end of the study; attendance was not reported) *Number of sessions:* 12 (45 min) *Therapist:* psychologist, nurses, occupational therapists, and social worker	Pre, post, follow-up+12, and follow-up+36 (36 months)	Depression symptoms (BDI-II) Anxiety symptoms (BAI)	Pain intensity (NRS) Fear avoidance (FABQ) Social functioning (SF-36-SR)
[5] Aragonès et al. (2019), Spain	Patients with CMP and MDD (measured with the SCID; cut-off was not reported), RCT	*Treatment arms:* CBT + TAU (*n* = 167) and TAU (*n* = 161) *Delivery period:* weekly (face-to-face)	*Intervention group*: CBT + TAU. Age: *M* = 61.4 (*SD* = 10.2). Gender: 138 females (82.6%) *Control group:* TAU. Age: *M* = 59.3 (*SD* = 10.1). Gender: 134 females (83.2%)	*Components:* optimized management of major depression, care management, and psychoeducation for chronic pain and depression (17% dropout rate at the end of the study and 49% attendance of at least 50% of the total number of sessions) *Number of sessions:* 9 (120 min) *Therapist:* psychologist and physician (primary care)	Pre, post, follow-up+6, and follow-up+12 (12 months)	Depression symptoms (HSCL-20)	Pain intensity (BPI) Pain interference (BPI)
[6] Boersma et al. (2019), Sweden	Patients with CMP and depression, and anxiety (measured with the HADS; cut-off ≥8 points), RCT	*Treatment arms:* CBT (*n* = 57) and Hybrid (*n* = 58) *Delivery period:* weekly (online)	*Intervention group*: CBT. Age: *M* = 45 (*SD* = 12). 44 (72.2) *Control group:* Hybrid. Age: *M* = 44 (*SD* = 12). Gender: 52 females (89.7%)	*Components:* CBT: psychoeducation (18% dropout rate at the end of the study and 30% attendance at least 75% of the total number of sessions); Hybrid: exposure *in vivo* and dialectical behavior therapy (DBT; 18% dropout rate at the end of the study and 65% attendance at least 75% of the total number of sessions) *Number of sessions:* 10–16 (75 min) *Therapist:* clinical psychologists and clinical psychologists in their post-graduate year	Pre, post, and follow-up+9 (9 months)	Depression symptoms (MADRS-S) Anxiety symptoms (GAD-7)	Pain catastrophizing (PCS) Pain intensity (MPI) Pain interference (MPI)
[7] Schlicker et al. (2020), Germany	Patients with CLBP and depression (measured with the CES-D; cut-off ≥16), RCT	*Treatment arms:* CBT + TAU (*n* = 40) and TAU (*n* = 36) *Delivery period:* weekly (online)	*Intervention group*: CBT + TAU. Age: *M* = 51.3 (*SD* = 8.6). Gender: 26 females (65%) *Control group:* TAU. Age: *M* = 50.1 (*SD* = 7.0). Gender: 29 females (81%)	*Components:* internet and mobile-based interventions based on CBT and visiting a general practitioner: psychoeducation, behavioral activation, and cognitive restructuring (35% dropout rate at the end of the study and 60% attendance of at least 80% of the total number of sessions) *Number of sessions:* 7 (45 to 60 min) *Therapist:* trained psychologists (eCoaches)	Pre, post, and follow-up+6 (6 months)	Depression symptoms (CES-D and QUIDS)	Anxiety (HADS-A) Quality of life (AQoL-6D and EQ-5D-5L) Social functioning (ODI-fd) Pain intensity (GPR) Pain self-efficacy (PSEQ) Working capacity (SPE)
[8] Baumeister et al. (2021), Germany	Patients with CBP and depression (measured with the SCID; cut-off was not reported), RCT	*Treatment arms:* CBT (*n* = 104) and TAU (*n* = 105) *Delivery period:* weekly (online)	*Intervention group*: CBT. Age: *M* = 50.3 (*SD* = 9.4). Gender: 60 females (58%) *Control group:* TAU. Age: *M* = 49.6 (*SD* = 9.3). Gender: 65 females (62%)	*Components:* internet and mobile program based on CBT: psychoeducation, behavior activation, and problem-solving (22 to 45%% dropout rate at the end of the study; attendance was not reported) *Number of sessions:* 6 regular and 3 optional (50 to 60 min) *Therapist:* trained psychologists (eCoaches)	Pre, post, and follow-up+6	Depression level (HPRSD)	Depression symptoms (PHQ-9) Pain intensity (NRS) Pain-related disability (ODI) Pain self-efficacy (PSEQ) Quality of Life (AQoL-6D) Work capacity (SPE)
[9] Gasslander et al. (2022), Sweden	Patients with CP and psychological distress (measured according to DSM-5), RCT	*Treatment arms:* CBT (*n* = 95) and TAU (*n* = 92) *Delivery period:* weekly (online)	*Intervention group*: CBT. Age: *M* = 45.6 (*SD* = 11.1). Gender: 70 females (74%) *Control group:* TAU. Age: *M* = 46.2 (*SD* = 11.2). Gender: 67 females (73%)	*Components:* internet program based on CBT: psychoeducation, relaxation, stress coping, behavioral activation, and maintenance (61% dropout rate at the end of the study and 35% attendance of at least 75% of the total number of sessions) *Number of sessions:* 6–13 (not reported) *Therapist:* psychologists	Pre, post, and follow-up+12 (12 months)*	Depression symptoms (MADR-S) Pain interference (MPI-S)	Depression and anxiety symptoms (HADS) Pain intensity (MPI-S) Pain acceptance (CPAQ) Coping strategies (CSQ-R) Pain catastrophizing (PCS) Quality of life (QoLI) Fear of anxiety symptoms (ASI) Social functioning (PDI) Pain self-efficacy (PSEQ-2) Kinesiophobia (TSK-11)
Mindfulness-based interventions (MBI)							
[10] De Jong et al. (2016, 2018), United States of America	Patients with CP and MDD (measured with the QIDS-C16; cut-off ≥6 points), pilot RCT	*Treatment arms:* MBCT + TAU (*n* = 26) and TAU (*n* = 14) *Delivery period:* weekly (face-to-face)	*Intervention group*: MBI + TAU. Age: *M* = 51.3 (*SD* = 11.9). Gender: 21 females (80.8%) *Control group:* TAU. Age: *M* = 49.9 (*SD* = 11.1). Gender: 9 females (64.3%)	*Components:* intervention based on MBI: CBT with a “mindful” approach (17% dropout rate at the end of the study and 73% attendance of at least 50% of the total number of sessions) *Number of sessions:* 8 (120 min) *Therapist:* clinical social worker (training in MBI)	Pre and post (2 months)	Depression symptoms (QIDS-C16 and HRSD17) Body awareness (MAIA)	Pain intensity (VAS) Pain interference (BPI) Anxiety (BAI) Quality of life (SF-36) Pain catastrophizing (PCS)
[11] Gardiner et al. (2019), United States of America	Patients with NSCP and MDD (measured with the PHQ-9; cut-off ≥5 points), RCT	*Treatment arms:* IMGV + TAU (*n* = 76) and TAU (*n* = 79) *Delivery period:* weekly (face-to-face and online)	*Intervention group*: IMGV + TAU. Age: *M* = 50 (*SD* = 12.2). Gender: 64 females (84%) *Control group:* TAU. Age: *M* = 51 (*SD* = 12.4). Gender: 70 females (89%)	*Components:* mindfulness techniques, evidence-based integrative medicine, and medical group visits (7% dropout rate at the end of the study and 72% attended at least 50% of the total number of sessions) *Number of sessions:* 9 (90 min) *Therapist:* physician and a co-facilitator with training in mindfulness	Pre, post, and follow-up+6 (5 months and 1 week)	Pain intensity (BPI) Depression level (PHQ-9)	Pain self-efficacy (PSEQ) Quality of life (SF-12) Behavioral activation (PAM)
[12] Torrijos-Zarcero et al. (2021), Spain	Patients with CP and depression and anxiety (measured with the HADS; cut-off ≥8 points), RCT	Treatment arms: MSC (*n* = 62) and CBT (*n* = 61) *Delivery period:* weekly (face-to-face)	*Intervention group*: MSC. Age: *M* = 48.29 (*SD* = 10.17). Gender: 56 females (90.3%) *Control group:* CBT. Age: *M* = 49.25 (*SD* = 11.39). Gender: 52 females (85.2%)	*Components:* MSC: formal meditation together with formal and informal self-compassion practices (33% dropout rate at the end of the study; adherence rate was not reported); and CBT: psychoeducation, relaxation, and cognitive restructuring (23% dropout rate; adherence rate was not reported) *Number of sessions:* 8 (150 min) *Therapist:* MSC: psychiatrist and art therapist (trained); and CBT: clinical psychologists (trained)	Pre and post	Self-compassion (SCS)	Pain interference (BPI) Pain intensity (PVAS) Anxiety and depression symptoms (HADS) Quality of life (SF-36) Pain catastrophizing (PCS) Pain acceptance (CPAQ)
Acceptance and Commitment Therapy (ACT) and Behavioral Activation Therapy (BATD)							
[13] Sanabria-Mazo et al. (2023), Spain	Patients with CLBP and depression (measured with the PHQ-9; cut-off ≥10 points), RCT	Treatment arms: ACT+TAU (*n* = 78), BATD+TAU (*n* = 78), and TAU (*n* = 78) *Delivery period:* weekly (online)	*Intervention groups*: ACT+TAU. Age: *M* = 54.9 (*SD* = 8.3). Gender: 54 females (69.2%). BATD+TAU. Age: *M* = 54.9 (*SD* = 10.2). Gender: 53 females (67.9%). *Control group:* TAU. Age: *M* = 53.8 (*SD* = 10.0). Gender: 51 females (65.4%)	*Components:* ACT+TAU (67% dropout rate at the end of the post-treatment and 56% at the end of the 12-month follow-up; and 53% attended at least 6 of the 8 sessions); and BATD+TAU (54% dropout rate at the end of the post-treatment and 50% at the end of the 12-months follow-up; and 46% attended at least 6 of the 8 sessions) *Number of sessions:* 8 (90 min) *Therapist:* ACT and BATD: clinical psychologists (trained)	Pre, post, during, and follow-up (12 months)	Pain interference (BPI)	Pain intensity (NRS) Depression, anxiety, and stress (DASS-21) Pain catastrophizing (PCS) Pain acceptance (CPAQ) Behavioral activation (BADS-SF) Psychological inflexibility (PIPS)

ADS, Allgemeine Depressions-Skala (German version of the CES-D); ACT, Acceptance and Commitment Therapy; ASI, Anxiety Sensitivity Index; AQoL-6D, Assessment of Quality of Life; BAI, Beck Anxiety Inventory; BAD-SF, Behavioral Activation for Depression Scale-Short; BATD, Behavioral Activation Therapy for Depression; BDI-II, Beck Depression Inventor; BPI, Brief Pain Inventory; CBP, chronic back pain; CBT, Cognitive Behavior Therapy; CES-D, Centre for Epidemiological Studies–Depression; CLBP, chronic low back pain; CMP, chronic musculoskeletal pain; COMM, Risk of Opioid Misuse; CP, chronic pain; CPAQ, Chronic Pain Acceptance Questionnaire; CSQ, Coping Strategies Questionnaire; DASS-21, Depression Anxiety Stress Scale; DSM-5, Diagnostic and Statistical Manual of Mental Disorders-5; EQ-5D-5L, EuroQol; FABQ, Fear-Avoidance Beliefs Questionnaire; GAD-7, Generalized Anxiety Disorder; GPR, Global Pain Rating; HADS, Hospital Anxiety and Depression Scale; HRSD, Hamilton Rating Scale for Depression; HSCL-20, Hopkins Symptom Checklist; IMGV, integrative medical group visits; MADRS-S, Montgomery–Åsberg Depression Rating Scale; MADRS-S, Montgomery–Åsberg Depression Rating Scale; MAIA, Multidimensional Assessment of Interceptive Awareness; MBCT, Mindfulness-Based Cognitive Therapy; MBI, mindfulness-based intervention; MDD, major depression disorder; MPI, Multidimensional Pain Inventory; MSC, mindful self-compassion; NNT, numbers needed to treat; NRS, Numerical Pain Rating Scale; NSCP, non-specific chronic pain; ODI-fd, Oswestry Disability Index; PAM, Patient Activation Measure; PCS, Pain Catastrophizing Scale; PDI, Pain Disability Index; PHQ-9, Patient Health Questionnaire-9; PIPS, Psychological Inflexibility in Pain Scale; PSEQ, Pain Self-Efficacy Questionnaire; PVAS, Pain Visual Analogue Scale; PWIA, Personal Well-being Index–Adult; QIDS, Quick Inventory of Depressive Symptomatology; QIDS-C16, Quick Inventory of Depressive Symptomatology–Clinician rated for DSM-IV; QoLI, Quality of Life Inventory; SCID, Structured Clinical Interview for DSM-IV; SF-12, 12-Item Short-Form Health Survey; SF-36, 36-Item Short-Form Health Survey; SPE, Subjective Prognosis of Employment Scale; TAU, treatment-as-usual; TSK-1, Tampa Scale of Kinesiophobia; VAS, visual analogue scale. *This study reported the between-group difference in the post-treatment comparison and intra-group difference in the pre-post-treatment and pre-follow-up + 12 comparison. Considering the objectives of this systematic review, only between-group comparisons are reported in this study.

### 3.3 Risk of bias assessment

**Figure 2:**
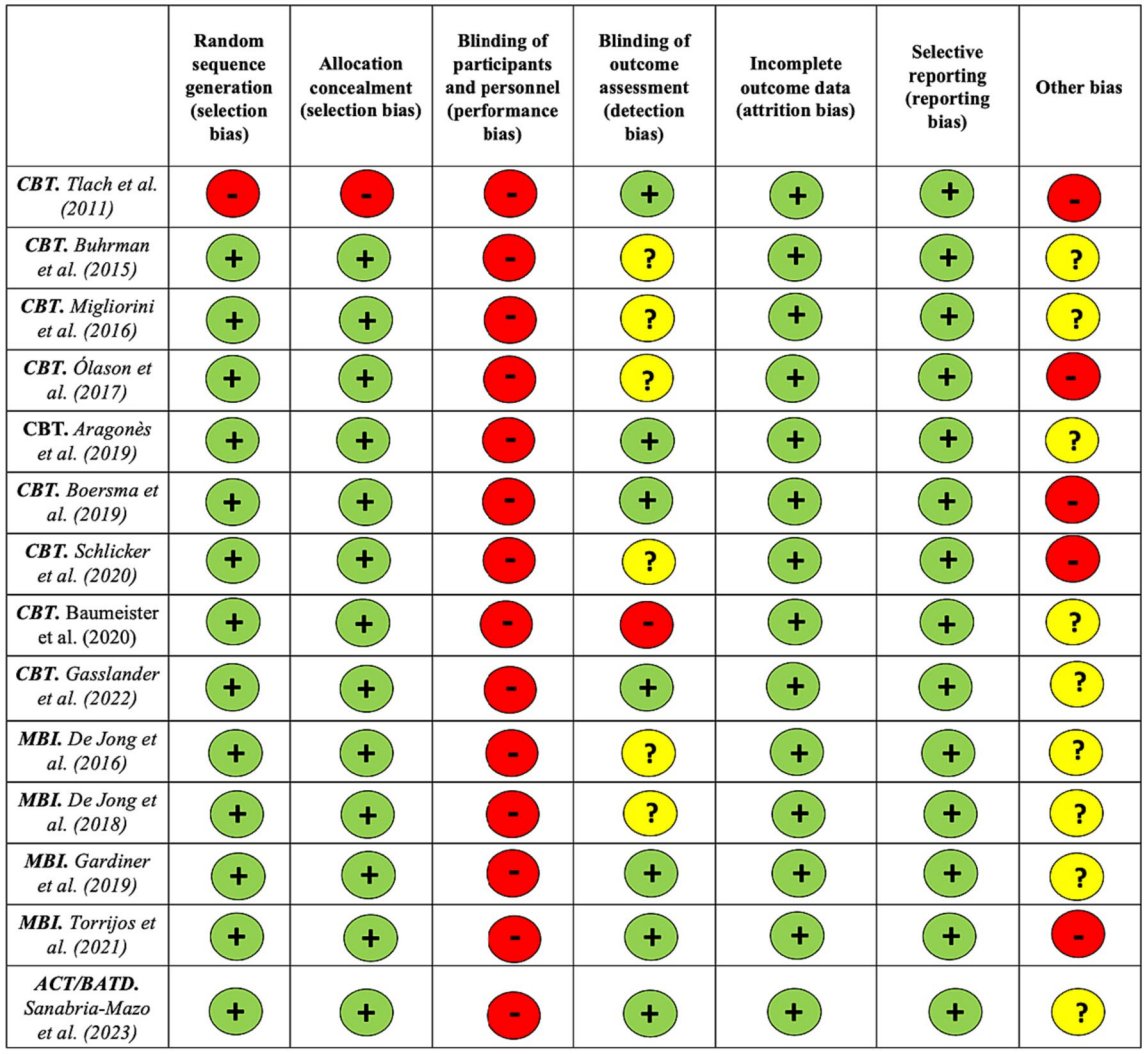
Risk of bias assessment for each included study using the Cochrane Collaboration’s tool for assessing risk of bias (Higgins et al., 2011).

Figure 2 shows the RoB for each included study. Twelve studies (92%) reported an adequate random sequence generation and provided sufficient information on the method of allocation concealment of patients. None of the studies (0%) blinded the participants and personnel to the intervention delivered. However, seven studies (54%) explicitly reported that they were able to blind outcome assessment from knowledge of which intervention a participant received. Incomplete outcome data were adequately managed in all cases (100%), and they were rated as free from selective outcome reporting bias in all included studies (100%). Considering the impossibility of blinding participants in psychological therapies, six studies (46%) reported a high (Tlach and Hampel, 2011; Ólason et al., 2018; Boersma et al., 2019; Schlicker et al., 2020; Baumeister et al., 2021; Torrijos-Zarcero et al., 2021) and seven (54%) an unclear RoB (Buhrman et al., 2015; De Jong et al., 2016, 2018; Migliorini et al., 2016; Aragonès et al., 2019; Gardiner et al., 2019; Gasslander et al., 2022; Sanabria-Mazo et al., 2023).

### 3.4 Psychological therapies

The specific results of each of the studies included in this systematic review are presented in Supplementary Table S3. Information from these controlled trials is organized according to the type of intervention (CBT, MBI, ACT, and BATD).

#### 3.4.1 Cognitive behavioral therapy (CBT)

Five out of the nine studies evaluated CBT as the only therapeutic component therapy (Tlach and Hampel, 2011; Ólason et al., 2018; Boersma et al., 2019; Baumeister et al., 2021; Gasslander et al., 2022) and the remaining four with other components (Buhrman et al., 2015; Migliorini et al., 2016; Aragonès et al., 2019; Schlicker et al., 2020). The time horizon of the assessment of eight out of the nine studies was pre-, post, and follow-up. Except for Tlach and Hampel (2011), Migliorini et al. (2016), and Ólason et al. (2018), all analyses of CBTs were based on ITT. Baseline comparisons were carried out in all nine CBT studies. Less Boersma et al. (2019), all studies compared CBT with an inactive control group (usual care).

All nine studies assessed depressive symptoms as the primary outcome (Buhrman et al., 2015; Migliorini et al., 2016; Ólason et al., 2018; Aragonès et al., 2019; Boersma et al., 2019; Schlicker et al., 2020; Baumeister et al., 2021; Gasslander et al., 2022) and three anxiety symptoms as the co-primary outcome (Tlach and Hampel, 2011; Buhrman et al., 2015; Ólason et al., 2018; Boersma et al., 2019). The characteristics of the CBT are detailed in Table 3 and the specific results of each study are presented in Supplementary Table S3. The evidence for each outcome is presented below.

##### 3.4.1.1 Depression

Six out of eight studies (75%) found significant differences in the reduction of depressive symptoms at post-treatment with very large to very small effect sizes (*d* ranging from 1.31 to 0.18; Tlach and Hampel, 2011; Buhrman et al., 2015; Migliorini et al., 2016; Schlicker et al., 2020; Baumeister et al., 2021; Gasslander et al., 2022); and four out of six studies (66%) at follow-up with medium to small effect sizes (*d* ranging from 0.75 to 0.26; Tlach and Hampel, 2011; Ólason et al., 2018; Aragonès et al., 2019; Baumeister et al., 2021) in favor of CBT compared to treatment as usual (TAU).

Another study (Boersma et al., 2019) identified significant differences in the reduction of depressive symptoms at follow-up with a small effect size (*d* = 0.25) in favor of hybrid therapy (exposure *in vivo* and DBT) compared to CBT.

##### 3.4.1.2 Anxiety

Five out of six studies (83%) also showed significant differences in the reduction of anxiety symptoms at post-treatment with large to very small effect sizes (*d* ranging from 1.08 to 0.19; Tlach and Hampel, 2011; Buhrman et al., 2015; Migliorini et al., 2016; Schlicker et al., 2020; Gasslander et al., 2022); and three out of four studies (75%) at follow-up with large to small effect sizes (*d* ranging from 1.07 to 0.27; Tlach and Hampel, 2011; Buhrman et al., 2015; Schlicker et al., 2020) in favor of CBT compared to TAU. No significant differences (0%) between these groups were found at post-treatment in two studies (Buhrman et al., 2015; Gasslander et al., 2022) and at follow-up in one study (Buhrman et al., 2015) exploring the fear of anxiety symptoms.

No significant differences (Boersma et al., 2019) were identified between CBT and hybrid therapy (exposure *in vivo* and DBT) in the reduction of anxiety symptoms at post-treatment and at follow-up.

##### 3.4.1.3 Stress

One out of one study (100%) identified significant differences in improved stress symptoms at follow-up with a small effect size (*d* = 0.47) in favor of CBT compared to TAU (Migliorini et al., 2016).

##### 3.4.1.4 Pain intensity

Significant differences in improved pain intensity were identified at post-treatment in one out of four studies with a small effect size (*d* = 0.42; Baumeister et al., 2021) in favor of CBT compared to TAU. No differences at follow-up were found in any of the four studies exploring pain intensity (Migliorini et al., 2016; Ólason et al., 2018; Aragonès et al., 2019; Schlicker et al., 2020).

Similarly, no significant differences were also found in the study (Boersma et al., 2019) comparing pain intensity after CBT and hybrid therapy (exposure *in vivo* and DBT) at post-treatment and follow-up.

##### 3.4.1.5 Pain interference

Two out of three studies (67%) found significant differences in the reduction of pain interference at post-treatment with small to very small (*d* ranging from 0.22 to 0.12; Buhrman et al., 2015; Gasslander et al., 2022), but not at the follow-up in the two studies (0%) that explored this outcome (Buhrman et al., 2015; Aragonès et al., 2019), in favor of the CBT compared to TAU.

Another study (Boersma et al., 2019) demonstrated significant changes in the reduction of pain interference in hybrid therapy (exposure *in vivo* and dialectical behavior therapy) compared to CBT at post-treatment with very small effect size (*d* = 0.02) and at follow-up with small effect size (*d* = 0.25).

##### 3.4.1.6 Pain catastrophizing

No significant differences (0%) between CBT and TAU were found at post-treatment in two studies (Buhrman et al., 2015; Gasslander et al., 2022) and at follow-up in one study (Buhrman et al., 2015) exploring pain catastrophizing.

However, another study (Boersma et al., 2019) reported significant differences in the decrease of pain catastrophizing at post-treatment with a small effect size (*d* = 0.26), but not at follow-up, in favor of hybrid therapy (exposure *in vivo* and dialectical behavior therapy) compared to CBT.

##### 3.4.1.7 Pain acceptance

Two out of two studies (100%) indicated significant differences in increased pain acceptance at post-treatment (Buhrman et al., 2015; Gasslander et al., 2022) with very small (*d* = 0.12) and small effect size (*d* = 0.30), but not at follow-up in one out of one study (0%) that explored this outcome, in favor of CBT compared to TAU.

##### 3.4.1.8 Pain self-efficacy

Significant differences between CBT and TAU were found at post-treatment in one out of three studies (33%) with a small effect size (*d* = 0.39; Baumeister et al., 2021) and at follow-up in one out of two studies (50%) with small effect size (*d* = 0.33; Baumeister et al., 2021).

No significant differences (0%) between CBT and TAU were found post-treatment in two studies (Schlicker et al., 2020; Gasslander et al., 2022) and at follow-up in one study (Schlicker et al., 2020) exploring pain self-efficacy.

##### 3.4.1.9 Quality of life

Four out of six studies (67%) found significant differences in improving quality of life at post-treatment with medium to invaluable effect sizes (*d* ranging from 0.78 to 0.02; Tlach and Hampel, 2011; Migliorini et al., 2016; Baumeister et al., 2021; Gasslander et al., 2022) and two out of four studies (50%) at follow-up with medium to small effect size (*d* = 0.78 and *d* = 0.33; Tlach and Hampel, 2011 and Baumeister et al., 2021, respectively) in favor of CBT compared to TAU.

##### 3.4.1.10 Social functioning

One out of one study (100%) identified significant differences in improved social functioning at follow-up with a medium effect size (*d* = 0.51) in favor of CBT compared to TAU (Ólason et al., 2018). No differences were found between these groups at post-treatment in the three studies (Ólason et al., 2018; Schlicker et al., 2020; Gasslander et al., 2022) exploring this outcome.

##### 3.4.1.11 Other outcomes

One out of two studies (50%) indicated significant differences in coping strategy of ignoring and catastrophizing at post-treatment (Gasslander et al., 2022) with small effect sizes (*d* = 0.38 and *d* = 0.34), but not at follow-up in one out of one study (0%) that explored cognitive and behavioral coping strategies (Buhrman et al., 2015), in favor of CBT compared to TAU. One out of one study (100%) identified significant differences in improved pain-related disability at post-treatment with a small effect size (*d* = 0.35) in favor of CBT compared to TAU (Baumeister et al., 2021), but not at follow-up.

No differences were found between CBT and TAU in one out of one study examining kinesiophobia (Gasslander et al., 2022), fear avoidance (Ólason et al., 2018), and life control (Gasslander et al., 2022) at post-treatment. Two studies explored work capacity at post-treatment and follow-up (Schlicker et al., 2020; Baumeister et al., 2021), but neither found significant differences (0%).

#### 3.4.2 Mindfulness-based interventions (MBI)

One of the MBI assessed the effects of Mindfulness-Based Cognitive Therapy (MBCT; De Jong et al., 2016, 2018), one of Integrative Medicine Group Visits (IMGV) with mindfulness techniques (Gardiner et al., 2019), and one of Mindful Self-Compassion (MSC) program (Torrijos-Zarcero et al., 2021). Two out of three studies evaluated MBI as the only therapeutic component (De Jong et al., 2016, 2018; Torrijos-Zarcero et al., 2021) and the remaining one as a multi-component (Gardiner et al., 2019) integrating mindfulness techniques, evidence-based integrative medicine, and medical group visits (Gardiner et al., 2019). The time horizon of the assessment of two of these studies was pre- and post (De Jong et al., 2016, 2018; Torrijos-Zarcero et al., 2021). All the analyses of MBIs were based on ITT. The efficacy of one study was tested in one RCT with results reported in two different publications (De Jong et al., 2016, 2018). Baseline comparisons were carried out in all MBI studies. Except for Torrijos-Zarcero et al. (2021), all studies compared MBI with an inactive control group (TAU).

Two studies assessed depressive symptoms as the primary outcome (De Jong et al., 2016, 2018; Gardiner et al., 2019); and one evaluated self-compassion (Torrijos-Zarcero et al., 2021) as the primary outcome and depressive and anxiety symptoms as the secondary outcome. The characteristics of the MBI are detailed in Table 3 and the specific results of each study are presented in Supplementary Table S3. The evidence for each outcome is detailed below.

##### 3.4.2.1 Depression

One study out of two (50%) identified significant differences in the reduction of depressive symptoms at post-treatment (De Jong et al., 2016, 2018) with a very small effect size (*d* = 0.13) in favor of MBI compared to TAU. The only study (Gardiner et al., 2019) that assessed depressive symptoms at follow-up found no significant difference between MBI and TAU.

No significant differences were identified in the study (Torrijos-Zarcero et al., 2021) comparing depressive symptoms at post-treatment between MBI and CBT.

##### 3.4.2.2 Anxiety

No differences were found between CBT and TAU in one out of one study examining anxiety symptoms at post-treatment and at follow-up (De Jong et al., 2016, 2018).

In contrast, one study (Torrijos-Zarcero et al., 2021) reported significant differences in the reduction of anxiety symptoms at post-treatment with a very small effect size (*d* = 0.17) in favor of MBI compared to CBT.

##### 3.4.2.3 Pain intensity

No significant differences between MBI and TAU (De Jong et al., 2016, 2018) and MBI and CBT (Torrijos-Zarcero et al., 2021) were reported at post-treatment in the reduction of pain intensity.

##### 3.4.2.4 Pain interference

Neither of the two studies comparing pain interference between MBI and TAU at post-treatment (De Jong et al., 2016, 2018; Gardiner et al., 2019) and at follow-up (Gardiner et al., 2019) showed significant differences.

However, one study (Torrijos-Zarcero et al., 2021) indicated significant differences in the reduction of pain interference at post-treatment with a very small effect size (*d* = 0.07) in favor of MBI compared to CBT.

##### 3.4.2.5 Pain catastrophizing

There was also no significant difference in the comparison between MBI and TAU in the reduction of pain catastrophizing in the only study (De Jong et al., 2016, 2018) that explored it at post-treatment.

One study (Torrijos-Zarcero et al., 2021) reported significant differences in decreasing pain catastrophizing at post-treatment with a very small effect size (*d* = 0.12) in favor of MBI compared to CBT.

##### 3.4.2.6 Pain acceptance

One study (Torrijos-Zarcero et al., 2021) reported significant differences in increasing pain acceptance at post-treatment with a very small effect size (*d* = 0.19) in favor of MBI compared to CBT.

##### 3.4.2.7 Pain self-efficacy

No significant differences between MBI and TAU (De Jong et al., 2016, 2018) were reported at post-treatment and follow-up in the reduction of pain self-efficacy.

##### 3.4.2.8 Quality of life

One study (De Jong et al., 2016, 2018) out of two found significant differences in improving quality of life at post-treatment with a very small effect size (*d* = 0.19); and one (Gardiner et al., 2019), the only one featuring this comparison, found a significant effect at follow-up (*RR = 1.07*) in favor of MBI compared to TAU.

In contrast, no significant differences in quality-of-life improvement were identified (Torrijos-Zarcero et al., 2021) between MBI and CBT.

##### 3.4.2.9 Mindfulness

One study (De Jong et al., 2016, 2018) showed significant differences in increased self-regulation with a large effect size (*d* = 0.91) and emotional awareness with a medium effect size (*d* = 0.57) at post-treatment.

Another study (Torrijos-Zarcero et al., 2021) identified significant differences in self-compassion with a very small effect size (*d* = 0.05) at post-treatment in favor of the MBI compared to CBT.

##### 3.4.2.10 Behavioral activation

No significant differences between MBI and TAU (De Jong et al., 2016, 2018) were reported at post-treatment and follow-up in the reduction of behavioral activation.

#### 3.4.3 Acceptance and commitment therapy (ACT) and behavioral activation therapy for depression (BATD)

One study explored the efficacy of ACT and BATD compared to TAU (Sanabria-Mazo et al., 2023). The time horizon of the assessment of this study was pre-, post, and follow-up and the analyses were based on ITT. Baseline comparisons were carried out in this study. This study assessed pain interference as the primary outcome. The characteristics of the ACT and BATD are detailed in Table 3 and the specific results of these studies are presented in Supplementary Table S3. The evidence for each outcome is detailed below.

##### 3.4.3.1 Depression, anxiety, and stress

Significant differences were detected in the improvement of stress symptoms at post-treatment with medium effect size (*d* = 0.69), but not at follow-up, in favor of ACT compared to TAU. However, no significant differences between these groups were found in depressive and anxiety symptoms. Similarly, no significant differences between BATD and TAU and between ACT and TAU were found in the improvement of depressive, anxiety, and stress symptoms.

##### 3.4.3.2 Pain interference, pain intensity, and pain catastrophizing

Significant differences between ACT and TAU were identified in the improvement of pain interference at post-treatment with a medium effect size (*d* = 0.64) and at follow-up with a medium effect size (*d* = 0.73). BATD was only statistically superior to TAU at follow-up with a medium effect size (*d* = 0.66). No significant differences between ACT and TAU, between BATD and TAU, and between ACT and BATD were found in pain intensity. A significant reduction in pain catastrophizing was reported by patients assigned to ACT and BATD at post-treatment with small and medium effect sizes (*d* = 0.45 and *d* = 0.59, respectively) and at follow-up with medium effect sizes (*d* = 0.59, in both) compared to TAU.

##### 3.4.3.3 Pain acceptance

Significant differences were found in the improvement of pain acceptance at post-treatment with a small effect size (*d* = 0.34) and at follow-up with a small effect size (*d* = 0.42) in ACT compared to TAU. In contrast, no significant differences between BATD and TAU and between ACT and BATD were found in pain acceptance.

##### 3.4.3.4 Psychological flexibility

Significant differences were identified in the improvement of psychological flexibility at post-treatment with a medium effect size (*d* = 0.52) and at follow-up with a small effect size (*d* = 0.37) in ACT compared to TAU. Similarly, significant differences between BATD and TAU were found in psychological flexibility with a small effect size (*d* = 0.40), but not at follow-up. No significant differences between ACT and BATD were found in psychological flexibility.

##### 3.4.3.5 Behavioral activation

Significant differences between ACT and TAU and between BATD and TAU were found in behavioral activation at post-treatment with small effect sizes (*d* = 0.30 and *d* = 0.46, respectively), but not at follow-up. No significant differences between ACT and BATD were found in behavioral activation.

### 3.5 Summary of results

Table 4 details a synthesis of all the evidence identified in the comparison between CBT, MBI, ACT, or BATD and TAU.

**Table 4:** Synthesis of all evidence identified in the comparison between CBT or MBI and TAU.

Outcome	Studies (*n*)	IG (*n*)	CG (*n*)	Significant* differences at posttreatment IG vs. CG (*n*, %)	Significant* differences at follow-up IG vs. CG (*n*, %)
Cognitive behavioral therapy (CBT)					
Depression	8 ^[1–5,7–9]^	551	521	(6/8, 75%) ^[1–3,7–9]^	(4/6, 67%) ^[1,4,5,8]^
Anxiety	6 ^[1–4,7,9]^	270	255	(5/6, 83%) ^[1,2,3,7,9]^	(3/4, 75%) ^[1,4,7]^
Stress	1 ^[3]^	34	25	(1/1, 100%) ^[3]^	–
Fear of anxiety	2 ^[2,9]^	123	116	(0/2, 0%)	(0/1, 0%)
Fear-avoidance	1 ^[4]^	39	38	(0/1, 0%)	–
Pain intensity	5 ^[4,5,7–9]^	445	432	(1/5, 20%) ^[8]^	(0/4, 0%)
Pain interference	4 ^[2,5,9]^	290	277	(2/3, 67%) ^[2,9]^	(0/2, 0%)
Pain catastrophizing	2 ^[2,9]^	123	116	(0/2, 0%)	(0/1, 0%)
Pain acceptance	2 ^[2,9]^	123	116	(2/2, 100%) ^[2,9]^	(0/1, 0%)
Pain self-efficacy	3 ^[7–9]^	239	232	(1/3, 33%) ^[8]^	(1/2, 50%) ^[8]^
Pain related disability	1 ^[8]^	104	105	(1/1, 100%) ^[8]^	(0/1, 0%)
Kinesiophobia	1 ^[9]^	95	92	(0/1, 0%)	–
Coping strategy	2 ^[2,9]^	123	116	(1/2, 50%) ^[9]^	(0/1, 0%)
Life control	1 ^[9]^	95	92	(1/1, 100%) ^[9]^	-
Working capacity	2 ^[7,8]^	144	141	(0/2, 0%)	(0/2, 0%)
Quality of life	6 ^[1–3,7–9]^	345	322	(4/6, 67%) ^[1,3,8,9]^	(2/4, 50%) ^[1,8]^
Social functioning	3 ^[4,7,9]^	174	166	(0/3, 0%)	(1/1, 100%) ^[4]^
Mindfulness-based interventions (MBI)					
Depression	2 ^[10,11]^	102	93	(1/2, 50%) ^[10]^	(0/1, 0%)
Anxiety	1 ^[10]^	26	14	(0/1, 0%)	(0/1, 0%)
Pain intensity	2 ^[10,11]^	88	75	(0/2, 0%)	-
Pain interference	2 ^[10,11]^	102	93	(0/2, 0%)	(0/1, 0%)
Pain catastrophizing	1 ^[10]^	26	14	(0/1, 0%)	-
Pain self-efficacy	1 ^[10]^	26	14	(0/1, 0%)	(0/1, 0%)
Quality of life	2 ^[10,11]^	102	93	(1/2, 50%) ^[10]^	(1/1, 100%) ^[10]^
Self-regulation	1 ^[10]^	26	14	(1/1, 100%) ^[10]^	-
Emotional awareness	1 ^[10]^	26	14	(1/1, 100%) ^[10]^	-
Behavioral activation	1 ^[10]^	26	14	(0/1, 0%)	(0/1, 0%)
Acceptance and commitment therapy (ACT)					
Depression	1 ^[13]^	78	78	(0/1, 0%)	(0/1, 0%)
Anxiety	1 ^[13]^	78	78	(0/1, 0%)	(0/1, 0%)
Stress	1 ^[13]^	78	78	(1/1, 100%)	(0/1, 0%)
Pain intensity	1 ^[13]^	78	78	(0/1, 0%)	(0/1, 0%)
Pain interference	1 ^[13]^	78	78	(1/1, 100%)	(1/1, 100%)
Pain catastrophizing	1 ^[13]^	78	78	(1/1, 100%)	(1/1, 100%)
Pain acceptance	1 ^[13]^	78	78	(1/1, 100%)	(1/1, 100%)
Behavioral activation	1 ^[13]^	78	78	(1/1, 100%)	(0/1, 0%)
Psychological inflexibility	1 ^[13]^	78	78	(1/1, 100%)	(1/1, 100%)
Behavioral activation therapy for depression (BATD)					
Depression	1 ^[13]^	78	78	(0/1, 0%)	(0/1, 0%)
Anxiety	1 ^[13]^	78	78	(0/1, 0%)	(0/1, 0%)
Stress	1 ^[13]^	78	78	(0/1, 100%)	(0/1, 0%)
Pain intensity	1 ^[13]^	78	78	(0/1, 0%)	(0/1, 0%)
Pain interference	1 ^[13]^	78	78	(1/1, 100%)	(1/1, 100%)
Pain catastrophizing	1 ^[13]^	78	78	(1/1, 100%)	(1/1, 100%)
Pain acceptance	1 ^[13]^	78	78	(0/1, 100%)	(0/1, 100%)
Behavioral activation	1 ^[13]^	78	78	(1/1, 100%)	(0/1, 0%)
Psychological inflexibility	1 ^[13]^	78	78	(1/1, 100%)	(0/1, 0%)

This table presents the evidence obtained in the exclusive comparison between CBT, MBI, ACT, or BATD and TAU. The evidence from the studies of [6] Boersma et al. (2019), a comparison of CBT and hybrid therapy (exposure in vivo and DBT), and [12] Torrijos-Zarcero et al. (2021), a comparison of MSC and CBT, is indicated in the text. The numbering of the synthesized evidence is indicated in brackets. [1] Tlach and Hampel (2011), [2] Buhrman et al. (2015), [3] Migliorini et al. (2016), [4] Ólason et al. (2018), [5] Aragonès et al. (2019), [7] Schlicker et al. (2020), [8] Baumeister et al. (2021), [9] Gasslander et al. (2022), [10] De Jong et al. (2016, 2018), [11] Gardiner et al. (2019), [13] Sanabria-Mazo et al. (2023). *p < 0.05.

### 3.6 Upcoming RCT

One upcoming RCT was identified. This RCT will evaluate the efficacy of internet-delivered ACT and internet-delivered CBT compared to attention control in patients with chronic non-cancer pain and major depression (Bell et al., 2020). The general characteristics of this study are detailed in Supplementary Table S4.

## 4 Discussion

Depression and anxiety are among the most diagnosed mental health conditions in people with chronic pain. Identification of effective therapies is needed because of the poorer prognosis and higher therapy resistance entailed in comorbid pain and psychological distress compared to either condition considered alone. However, to date, no published systematic reviews have attempted to synthesize the efficacy of these interventions in patients with these combined conditions. The current systematic review demonstrates positive, but modest, results from CBT-based interventions for patients with chronic pain and clinically relevant psychological distress. A total of twelve RCTs and one non-RCT published between 2011 and 2023 were included in the analyses. In addition, it was noted that one RCT is upcoming that will explore the efficacy of ACT and traditional CBT in patients with chronic non-cancer pain and major depression, and results are expected soon (Bell et al., 2020). Taken together, the published and upcoming studies signal an increasing interest in examining how CBT-based therapies (CBT, MBI, ACT, and BATD) can improve the functional status and quality of life in patients with chronic pain experiencing clinically relevant depressive and/or anxiety symptoms. There is also an increasing interest in recognizing potential beneficial therapeutic processes of change in patients with this comorbidity in the second and third wave of CBTs (Hayes and Hofmann, 2021), such as acceptance of pain, psychological flexibility, and behavioral activation (Buhrman et al., 2015; Bell et al., 2020; Gasslander et al., 2022; Sanabria-Mazo et al., 2023).

Compared to TAU, traditional CBT reported significant differences in the reduction of depressive and anxiety symptoms and in the increase of quality of life at post-treatment and at follow-up, with very large to small effect sizes. These results are consistent with the reported efficacy of CBT-based interventions for depression or chronic pain in previous systematic reviews (Lorenzo-Luaces et al., 2018; López-López et al., 2019; Williams et al., 2020), but with a more modest magnitude. Nevertheless, in general, no significant differences between traditional CBT and TAU were identified at post-treatment and follow-up in the studies exploring pain intensity and pain catastrophizing. Although with a limited number of studies, there is also evidence that CBT could be beneficial in improving pain interference and pain acceptance (Buhrman et al., 2015; Gasslander et al., 2022) at posttreatment, but not at follow-up, with small effect sizes. In other pain-related variables, such as pain self-efficacy, pain-related disability, fear avoidance, kinesiophobia, working capacity, and social functioning, inconsistent results or insufficient evidence were obtained.

As in previous research in chronic pain (Veehof et al., 2016; Hilton et al., 2017; Khoo et al., 2019), compared to TAU, MBI produced a significant reduction at post-treatment in depressive symptoms, in one out of two studies (De Jong et al., 2018), and an increase in emotional awareness and self-regulation, in the one study that addressed this (De Jong et al., 2016). However, this evidence comes from a pilot RCT with a small sample size (De Jong et al., 2016, 2018). More evidence is needed to determine the overall efficacy of MBI in depression, anxiety, pain, and quality of life for populations with this comorbidity. Results from a single study (Torrijos-Zarcero et al., 2021) indicated significant differences in anxiety, pain interference, pain acceptance, pain catastrophizing, and self-compassion at post-treatment in favor of MBI compared to CBT.

Findings from a recent RCT provided evidence of the clinical utility of including remote synchronous video group-based ACT or BATD as adjuncts to TAU for the improvement of pain interference and pain catastrophizing after treatment and in the follow-up to patients with chronic low back pain (CLBP) and comorbid depressive symptoms. However, no significant differences in depressive or anxiety symptoms were found in ACT and BATD compared to TAU at any assessment time points. In both active therapies, improvements in pain interference at follow-up were significantly mediated by improvements at post-treatment in psychological flexibility (Sanabria-Mazo et al., 2023). Investigating the mediating role of psychological flexibility in the third wave of CBTs for chronic pain patients is important for understanding the mechanisms of change underlying treatment effectiveness, identifying effective treatment components, and enhancing treatment outcomes (McCracken et al., 2022). The results of the Bell et al. (2020) study, when available, could help provide stronger evidence for the findings known so far in the population with this comorbidity.

In most of the studies explored in this systematic review, CBT-based interventions were more effective than control groups in improving depression, anxiety, and quality of life, at both post-treatment and at follow-up, but not in the improvement of pain intensity. However, the findings of this systematic review should be interpreted with some caution, as they are based on few studies with high heterogeneity in terms of mode of delivery (e.g., face-to-face, online, and blended format), number of sessions, intervention components, compliance, and characteristics of therapists, among others. It is also important to consider the potential bias arising from studies with samples smaller than 50 participants per arm and the lack of information on the adverse effects of therapies (Moore et al., 2010). A recent Delphi study has pointed out the importance of recognizing what the main contents of CBT are. In this regard, three main components have been highlighted: (1) pain education; (2) increased activity; and (3) some form of cognitive challenge (Sharpe et al., 2020). In the studies included, there were also some differences in the types of CBT methods used or in the primary and secondary outcomes, which complicates the generalizability of these results.

Like previous meta-analyses in chronic pain (Williams et al., 2020) and depression (Lorenzo-Luaces et al., 2018), the efficacy of CBT-based interventions for comorbid pain and depression is clinically relevant on average (Sanabria-Mazo et al., 2020). As the findings of this study point out, the effects of CBT targeting the population with chronic pain and comorbid psychological distress are more modest than targeting one of the two conditions separately (Sanabria-Mazo et al., 2020). Psychological distress could potentially impact adherence to pain management interventions, leading to decreased engagement in self-care activities, and treatment plan compliance among patients with depression or anxiety, ultimately affecting treatment outcomes. Hence, it is crucial to evaluate and tackle depression in chronic pain populations for better treatment outcomes.

While the results of this systematic review fit with a wider conclusion that traditional CBT is beneficial for many varied conditions (Fordham et al., 2021), there appears substantial room for improvement. Considering the effects identified, it would be interesting to explore, when more robust evidence is available, the efficacy of third-generation therapies in patients with chronic pain and comorbid psychological stress. Although evidence is beginning to emerge on the effects of third-wave CBT therapies compared with TAU (De Jong et al., 2016, 2018; Gardiner et al., 2019; Bell et al., 2020; Torrijos-Zarcero et al., 2021; Sanabria-Mazo et al., 2023), more research is needed to compare which therapy is most effective, in which circumstances, and for whom.

### 4.1 Limitations and strengths

These findings must be interpreted to understand the following limitations and strengths. First, given the lack of trials with low RoB, it might be premature to conclude the magnitude of the efficacy of CBT-based interventions for this comorbidity. Second, since the heterogeneity of available data in the included studies (e.g., mode of delivery, number of sessions, intervention components, and characteristics of therapists, among others), it was not possible to compute a meta-analysis. Third, although published and unpublished studies were explored, only published studies in English or Spanish were finally included in this systematic review, so other otherwise relevant evidence could have been omitted. Fourth, due to the limited number of RCTs, it was not possible to examine whether specific forms of CBT are more effective than others. The strengths of this study are the number of databases explored, the compliance with PRISMA guidelines, the validation of the Boolean searches according to PRESS guidelines, the use of Rayyan as a tool to minimize possible loss of evidence, and the consensual review between reviewers in the different phases of screening, extraction of the data, and RoB.

### 4.2 Future research

Further research is needed in this area when more studies are available. The need to identify the core elements of psychosocial therapies that drive their therapeutic effects is critical. To extend the knowledge on the relevant topic examined in this study, future studies should explore the ingredients that are indeed effective and for which patients, as well as what amount of variance is explained by universal factors shared by all therapies. These interventions should also strive to employ adequately powered randomized designs and compare the efficacy of psychological therapies to other empirically supported therapies.

## 5 Conclusion

The comorbidity of chronic pain and psychological distress represents a complex problem or set of problems, perhaps best conceived as having a multifactorial aetiology. Psychological research and treatment should address these because when they appear together, they cause substantial health and social impacts. This study shows that traditional CBT improves depression, anxiety, and quality of life in patients with comorbid chronic pain and clinically relevant psychological distress, but not for pain intensity and pain catastrophizing. Although some evidence is presented in this systematic review, more RCTs based on MBI, ACT, and BATD are needed to determine the overall efficacy of this intervention in these patients.

## Data availability statement

The original contributions presented in the study are included in the article/Supplementary material, further inquiries can be directed to the corresponding author.

## Author contributions

JL, AS, SE, and JS-M designed the study. JS-M, AC-C, ÓF-V, and GN-R performed the eligibility criteria, data extraction, and study coding. JS-M and AC-C performed the data analysis and synthesized all extracted data. JS-M drafted the manuscript. GC-R, AM-P, JC-A, SE, XB, AS, AF-S, and JL revised and approved the final version of the manuscript. LM critically revised and supervised the final draft. All authors commented on, revised, and approved the draft and the final manuscript.

## References

[ref1] Ahern E. Kinsella S. Semkovska M. (2018). Clinical efficacy and economic evaluation of online cognitive behavioral therapy for major depressive disorder: a systematic review and meta-analysis. Expert Rev. Pharmacoecon. Outcomes Res. *18*, 25–41. doi: 10.1080/14737167.2018.1407245, PMID: 29145746

[ref2] Aragonès E. Rambla C. López-Cortacans G. Tomé-Pires C. Sánchez-Rodríguez E. Caballero A. . (2019). Effectiveness of collaborative care intervention for managing major depression and chronic musculoskeletal pain in primary care: a cluster-randomized controlled trial. J. Affect. Disord. *252*, 221–229. doi: 10.1016/j.jad.2019.04.004, PMID: 30986737

[ref3] Baumeister H. Knecht A. Hutter N. (2012). Direct and indirect costs in persons with chronic back pain and comorbid mental disorders: a systematic review. J. Psychosom. Res. *73*, 79–85. doi: 10.1016/j.jpsychores.2012.05.008, PMID: 22789408

[ref4] Baumeister H. Paganini S. Sander L. B. Lin J. Schlicker S. Terhorst Y. . (2021). Effectiveness of a guided internet-and mobile-based intervention for patients with chronic back pain and depression (WARD-BP): a multicenter, pragmatic randomized controlled trial. Psychother. Psychosom. *90*, 255–268. doi: 10.1159/000511881, PMID: 33321501

[ref5] Bell L. V. Cornish P. Flusk D. Garland S. N. Rash J. A. (2020). The INternet ThERapy for deprESsion trial (INTEREST): protocol for a patient-preference, randomised controlled feasibility trial comparing iACT, iCBT and attention control among individuals with comorbid chronic pain and depression. BMJ Open *10*:e033350. doi: 10.1136/bmjopen-2019-033350, PMID: 32114466 PMC7050318

[ref6] Bisby M. A. Chandra S. S. Dudeney J. Scott A. J. Titov N. Dear B. F. (2022). Can internet-delivered pain management programs reduce psychological distress in chronic pain? Exploring relationships between anxiety and depression, pain intensity, and disability. Pain Med. 24, 538–546. doi: 10.1093/pm/pnac158, PMID: 36315066

[ref7] Boersma K. Södermark M. Hesser H. Flink I. K. Gerdle B. Linton S. J. (2019). Efficacy of a transdiagnostic emotion–focused exposure treatment for chronic pain patients with comorbid anxiety and depression: a randomised controlled trial. Pain *160*, 1708–1718. doi: 10.1097/j.pain.0000000000001575, PMID: 31335641 PMC6687409

[ref8] Buhrman M. Gordh T. Andersson G. (2016). Internet interventions for chronic pain including headache: a systematic review. Internet Interv. *4*, 17–34. doi: 10.1016/j.invent.2015.12.001, PMID: 30135787 PMC6096254

[ref9] Buhrman M. Syk M. Burvall O. Hartig T. Gordh T. Andersson G. (2015). Individualized guided internet-delivered cognitive behavior therapy for chronic pain patients with comorbid depression and anxiety. Clin. J. Pain *31*, 504–516. doi: 10.1097/AJP.0000000000000176, PMID: 25380222

[ref10] Chopra K. Arora V. (2014). An intricate relationship between pain and depression: clinical correlates, coactivation factors, and therapeutic targets. Expert Opin. Ther. Targets *18*, 159–176. doi: 10.1517/14728222.2014.855720, PMID: 24295272

[ref11] Churchill R. Moore T. H. Furukawa T. A. Caldwell D. M. Davies P. Jones H. . (2013). 'Third wave' cognitive and behavioural therapies versus treatment as usual for depression. Cochrane Database Syst. Rev. 10:CD008705. doi: 10.1002/14651858.CD008705.pub2, PMID: 24142810 PMC13035257

[ref12] Cuijpers P. Berking M. Andersson G. Quigley L. Kleiboer A. Dobson K. S. (2013). A meta-analysis of cognitive-behavioural therapy for adult depression, alone and in comparison with other treatments. Can. J. Psychiatr. *58*, 376–385. doi: 10.1177/070674371305800702, PMID: 23870719

[ref13] De Jong M. Lazar S. W. Hug K. Mehling W. E. Hölzel B. K. Sack A. T. . (2016). Effects of mindfulness-based cognitive therapy on body awareness in patients with chronic pain and comorbid depression. Front. Psychol. *7*:967. doi: 10.3389/fpsyg.2016.00967, PMID: 27445929 PMC4927571

[ref14] De Jong M. Peeters F. Gard T. Ashih H. Doorley J. Walker R. . (2018). A randomised controlled pilot study on mindfulness-based cognitive therapy for unipolar depression in patients with chronic pain. J. Clin. Psychiatry *79*:15m10160. doi: 10.4088/JCP.15m10160, PMID: 28252881 PMC6020018

[ref15] Drapeau A. Marchand A. Beaulieu-Prévost D. (2012). “Epidemiology of psychological distress” in Mental illnesses – understanding, prediction and control. ed. Labate L. (London: IntechOpen Limited), 105–133.

[ref16] Dworkin R. H. Turk D. C. Wyrwich K. W. Beaton D. Cleeland C. S. Farrar J. T. . (2008). Interpreting the clinical importance of treatment outcomes in chronic pain clinical trials: IMMPACT recommendations. J. Pain *9*, 105–121. doi: 10.1016/j.jpain.2007.09.005, PMID: 18055266

[ref17] Fordham B. Sugavanam T. Edwards K. Stallard P. Howard R. Das-Nair R. . (2021). The evidence for cognitive behavioral therapy in any condition, population, or context: a meta-review of systematic reviews and panoramic meta-analysis. Psychol. Med. *51*, 21–29. doi: 10.1017/S0033291720005292, PMID: 33455594 PMC7856415

[ref18] Gardiner P. Luo M. D’Amico S. Gergen-Barnett K. White L. F. Saper R. . (2019). Effectiveness of integrative medicine group visits in chronic pain and depressive symptoms: a randomised controlled trial. PLoS One *14*:e0225540. doi: 10.1371/journal.pone.0225540, PMID: 31851666 PMC6919581

[ref19] Gasslander N. Andersson G. Boström F. Brandelius L. Pelling L. Hamrin L. . (2022). Tailored internet-based cognitive behavioral therapy for individuals with chronic pain and comorbid psychological distress: a randomized controlled trial. Cogn. Behav. Ther. *51*, 408–434. doi: 10.1080/16506073.2022.2065528, PMID: 35533363

[ref20] Gloster A. T. Walder N. Levin M. Twohig M. Karekla M. (2020). The empirical status of acceptance and commitment therapy: a review of meta-analyses. J. Contextual Behav. Sci. *18*, 181–192. doi: 10.1016/j.jcbs.2020.09.009

[ref21] Haugmark T. Hagen K. B. Smedslund G. Zangi H. A. (2019). Mindfulness-and acceptance-based interventions for patients with fibromyalgia–a systematic review and meta-analyses. PLoS One *14*:e0221897. doi: 10.1371/journal.pone.0221897, PMID: 31479478 PMC6719827

[ref22] Hayes S. C. Hofmann S. G. (2021). "Third-wave" cognitive and behavioral therapies and the emergence of a process-based approach to intervention in psychiatry. World Psychiatry *20*, 363–375. doi: 10.1002/wps.20884, PMID: 34505370 PMC8429332

[ref23] Higgins J. P. Altman D. G. Gøtzsche P. C. Jüni P. Moher D. Oxman A. D. . (2011). The Cochrane Collaboration’s tool for assessing risk of bias in randomised trials. BMJ *343*:d5928. doi: 10.1136/bmj.d5928, PMID: 22008217 PMC3196245

[ref24] Hilton L. Hempel S. Ewing B. A. Apaydin E. Xenakis L. Newberry S. . (2017). Mindfulness meditation for chronic pain: systematic review and meta-analysis. Ann. Behav. Med. *51*, 199–213. doi: 10.1007/s12160-016-9844-2, PMID: 27658913 PMC5368208

[ref25] Hooten W. M. (2016). Chronic pain and mental health disorders: shared neural mechanisms, epidemiology, and treatment. Mayo Clin. Proc. *91*, 955–970. doi: 10.1016/j.mayocp.2016.04.029 27344405

[ref26] Hughes L. S. Clark J. Colclough J. A. Dale E. McMillan D. (2017). Acceptance and commitment therapy (ACT) for chronic pain. Clin. J. Pain *33*, 552–568. doi: 10.1097/AJP.0000000000000425 27479642

[ref27] Jorn A. C. (2015). Elements of the biopsychosocial interview of the chronic pain patient: a new expanded model using rational emotive behavior therapy. J. Ration. Emot. Cogn. Behav. Ther. *33*, 284–307. doi: 10.1007/s10942-015-0217-8

[ref28] Khoo E. L. Small R. Cheng W. Hatchard T. Glynn B. Rice D. B. . (2019). Comparative evaluation of group-based mindfulness-based stress reduction and cognitive behavioral therapy for the treatment and management of chronic pain: a systematic review and network meta-analysis. Evid. Based Ment. Health *22*, 26–35. doi: 10.1136/ebmental-2018-300062, PMID: 30705039 PMC10270397

[ref29] Kroenke K. Wu J. Bair M. J. Krebs E. E. Damush T. M. Tu W. (2011). Reciprocal relationship between pain and depression: a 12-month longitudinal analysis in primary care. J. Pain *12*, 964–973. doi: 10.1016/j.jpain.2011.03.003, PMID: 21680251 PMC3222454

[ref30] Lin J. Scott W. Carpenter L. Norton S. Domhardt M. Baumeister H. . (2019). Acceptance and commitment therapy for chronic pain: protocol of a systematic review and individual participant data meta-analysis. Syst. Rev. *8*, 140–110. doi: 10.1186/s13643-019-1044-2, PMID: 31200768 PMC6570828

[ref31] López-López J. A. Davies S. R. Caldwell D. M. Churchill R. Peters T. J. Tallon D. . (2019). The process and delivery of CBT for depression in adults: a systematic review and network meta-analysis. Psychol. Med. *49*, 1937–1947. doi: 10.1017/S003329171900120X, PMID: 31179960 PMC6712954

[ref32] Lorenzo-Luaces L. Johns E. Keefe J. R. (2018). The generalizability of randomised controlled trials of self-guided internet-based cognitive behavioral therapy for depressive symptoms: systematic review and meta-regression analysis. J. Med. Internet Res. *20*:e10113. doi: 10.2196/10113, PMID: 30413400 PMC6251981

[ref33] Mansfield K. E. Sim J. Jordan J. L. Jordan K. P. (2016). A systematic review and meta-analysis of the prevalence of chronic widespread pain in the general population. Pain *157*, 55–64. doi: 10.1097/j.pain.0000000000000314, PMID: 26270591 PMC4711387

[ref34] McCracken L. M. (2023). Personalized pain management: is it time for process-based therapy for particular people with chronic pain? Eur. J. Pain 27, 1044–1055. doi: 10.1002/ejp.2091, PMID: 36755478

[ref35] McCracken L. M. Yu L. Vowles K. E. (2022). New generation psychological treatments in chronic pain. BMJ 376:e057212. doi: 10.1136/bmj-2021-057212 35228207

[ref36] McGowan J. Sampson M. Salzwedel D. M. Cogo E. Foerster V. Lefebvre C. (2016). PRESS peer review of electronic search strategies: 2015 guideline statement. J. Clin. Epidemiol. *75*, 40–46. doi: 10.1016/j.jclinepi.2016.01.021, PMID: 27005575

[ref37] Migliorini C. Sinclair A. Brown D. Tonge B. New P. (2016). A randomised control trial of an internet-based cognitive behaviour treatment for mood disorder in adults with chronic spinal cord injury. Spinal Cord *54*, 695–701. doi: 10.1038/sc.2015.221, PMID: 26690861

[ref38] Moore A. R. Eccleston C. Derry S. Wiffen P. Bell R. F. Straube S. . (2010). “Evidence” in chronic pain–establishing best practice in the reporting of systematic reviews. Pain *150*, 386–389. doi: 10.137110.1016/j.pain.2010.05.011, PMID: 20627575

[ref39] Ólason M. Andrason R. H. Jónsdóttir I. H. Kristbergsdóttir H. Jensen M. P. (2018). Cognitive behavioral therapy for depression and anxiety in an interdisciplinary rehabilitation program for chronic pain: a randomised controlled trial with a 3-year follow-up. Int. J. Behav. Med. *25*, 55–66. doi: 10.1007/s12529-017-9690-z, PMID: 29094283

[ref40] Page M. J. McKenzie J. E. Bossuyt P. M. Boutron I. Hoffmann T. C. Mulrow C. D. . (2021). The PRISMA 2020 statement: an updated guideline for reporting systematic reviews. Int. J. Surg. *88*:105906. doi: 10.1016/j.ijsu.2021.105906, PMID: 33789826

[ref41] Pardos-Gascón E. M. Narambuena L. Leal-Costa C. Van-der Hofstadt-Román C. J. (2021). Differential efficacy between cognitive-behavioral therapy and mindfulness-based therapies for chronic pain: systematic review. Int. J. Clin. Health Psychol. *21*:100197. doi: 10.1016/j.ijchp.2020.08.001, PMID: 33363580 PMC7753033

[ref42] Pasarelu C. R. Andersson G. Bergman Nordgren L. Dobrean A. (2017). Internet-delivered transdiagnostic and tailored cognitive behavioral therapy for anxiety and depression: a systematic review and meta-analysis of randomized controlled trials. Cogn. Behav. Ther. *46*, 1–28. doi: 10.1080/16506073.2016.1231219, PMID: 27712544

[ref43] Rayner L. Hotopf M. Petkova H. Matcham F. Simpson A. McCracken L. M. (2016). Depression in patients with chronic pain attending a specialised pain treatment Centre: prevalence and impact on health care costs. Pain *157*, 1472–1479. doi: 10.1097/j.pain.0000000000000542, PMID: 26963849 PMC4912238

[ref44] Reid K. J. Harker J. Bala M. M. Truyers C. Kellen E. Bekkering G. E. . (2011). Epidemiology of chronic non-cancer pain in Europe: narrative review of prevalence, pain treatments, and pain impact. Curr. Med. Res. Opin. 27, 449–462. doi: 10.1185/03007995.2010.545813, PMID: 21194394

[ref45] Roberts T. Esponda G. M. Krupchanka D. Shidhaye R. Patel V. Rathod S. (2018). Factors associated with health service utilisation for common mental disorders: a systematic review. BMC Psychiatry *18*, 1–19. doi: 10.1186/s12888-018-1837-1, PMID: 30134869 PMC6104009

[ref46] Sanabria-Mazo J. P. Colomer-Carbonell A. Borràs X. Castaño-Asins J. R. McCracken L. M. Montero-Marin J. . (2023). Efficacy of videoconference group acceptance and commitment therapy (ACT) and behavioral activation therapy for depression (BATD) for chronic low back pain (CLBP) and comorbid depressive symptoms: a randomized controlled trial (IMPACT study). J. Pain *24*, 1522–1540. doi: 10.1016/j.jpain.2023.04.008, PMID: 37105508

[ref47] Sanabria-Mazo J. P. Forero C. G. Cristobal-Narváez P. Suso-Ribera C. García-Palacios A. Colomer-Carbonell A. . (2020). Efficacy, cost-utility and physiological effects of acceptance and commitment therapy (ACT) and behavioral activation treatment for depression (BATD) in patients with chronic low back pain and depression: study protocol of a randomised, controlled trial including mobile-technology-based ecological momentary assessment (IMPACT study). BMJ Open *10*:e038107. doi: 10.1136/bmjopen-2020-038107, PMID: 32709656 PMC7380881

[ref48] Sawilowsky S. S. (2009). New effect size rules of thumb. J. Mod. Appl. Stat. Methods *8*, 597–599. doi: 10.22237/jmasm/1257035100

[ref49] Schlicker S. Baumeister H. Buntrock C. Sander L. Paganini S. Lin J. . (2020). A web-and mobile-based intervention for comorbid, recurrent depression in patients with chronic back pain on sick leave (get. back): pilot randomised controlled trial on feasibility, user satisfaction, and effectiveness. JMIR Ment. Health *7*:e16398. doi: 10.2196/16398, PMID: 32293577 PMC7191351

[ref50] Sharpe L. Jones E. Ashton-James C. E. Nicholas M. K. Refshauge K. (2020). Necessary components of psychological treatment in pain management programs: a Delphi study. Eur. J. Pain 24, 1160–1168. doi: 10.1002/ejp.1561, PMID: 32187442

[ref51] Snyder M. Handrup C. T. (2018). Challenges in treatment of comorbid chronic pain, depression, and anxiety. J. Psychosoc. Nurs. Ment. Health Serv. *56*, 17–21. doi: 10.3928/02793695-20180601-01, PMID: 29916524

[ref52] Tlach L. Hampel P. (2011). Long-term effects of a cognitive-behavioral training program for the management of depressive symptoms among patients in orthopedic inpatient rehabilitation of chronic low back pain: a 2-year follow-up. Eur. Spine J. *20*, 2143–2151. doi: 10.1007/s00586-011-1810-x, PMID: 21516327 PMC3229734

[ref53] Torrijos-Zarcero M. Mediavilla R. Rodríguez-Vega B. Del Río-Diéguez M. López-Álvarez I. Rocamora-González C. . (2021). Mindful self-compassion program for chronic pain patients: a randomised controlled trial. Eur. J. Pain 25, 930–944. doi: 10.1002/ejp.1734, PMID: 33471404

[ref54] Veehof M. M. Trompetter H. R. Bohlmeijer E. T. Schreurs K. M. G. (2016). Acceptance-and mindfulness-based interventions for the treatment of chronic pain: a meta-analytic review. Cogn. Behav. Ther. *45*, 5–31. doi: 10.1080/16506073.2015.1098724, PMID: 26818413

[ref55] Walker A. K. Kavelaars A. Heijnen C. J. Dantzer R. (2014). Neuroinflammation and comorbidity of pain and depression. Pharmacol. Rev. *66*, 80–101. doi: 10.1124/pr.113.008144, PMID: 24335193 PMC3880465

[ref56] White V. Linardon J. Stone J. E. Holmes-Truscott E. Olive L. Mikocka-Walus A. . (2022). Online psychological interventions to reduce symptoms of depression, anxiety, and general distress in those with chronic health conditions: a systematic review and meta-analysis of randomized controlled trials. Psychol. Med. *52*, 548–573. doi: 10.1017/S0033291720002251, PMID: 32674747

[ref57] Williams A. C. C. Fisher E. Hearn L. Eccleston C. (2020). Psychological therapies for the management of chronic pain (excluding headache) in adults. Cochrane Database Syst. Rev. *2021*:CD007407. doi: 10.1002/14651858.CD007407.pub4, PMID: 32794606 PMC7437545

[ref58] Wittchen H. U. Jacobi F. Rehm J. Gustavsson A. Svensson M. Jönsson B. . (2011). The size and burden of mental disorders and other disorders of the brain in Europe 2010. Eur. Neuropsychopharmacol. *21*, 655–679. doi: 10.1016/j.euroneuro.2011.07.018, PMID: 21896369

