# Supplementary material for: A systematic review of cognitive behavioral therapy-based interventions for comorbid chronic pain and clinically relevant psychological distress
Source: Front Psychol. 2023 Dec 22;14:1200685. doi: 10.3389/fpsyg.2023.1200685 (PMC10766814; doi:10.3389/fpsyg.2023.1200685)
Supplement: Supplementary file 1 [file Data_Sheet_1.docx]

Supplementary Material

A systematic review of cognitive behavioral therapy-based interventions for comorbid chronic pain and clinically relevant psychological distress

Juan P. Sanabria-Mazo, Ariadna Colomer-Carbonell, Óscar Fernández-Vázquez, Georgina Noboa-Rocamora, Gemma Cardona-Ros, Lance M. McCracken, Antonio Montes-Pérez, Juan R. Castaño-Asins, Sílvia Edo, Xavier Borràs, Antoni Sanz, Albert Feliu-Soler and Juan V. Luciano*

*** Correspondence:**

Juan V. Luciano
[juanvicente.luciano@uab.cat](mailto:juanvicente.luciano@uab.cats)

# Supplementary Table 1

## Protocol adaptations

Some changes have been made since the publication of the protocol, such as the incorporation of two new expert coauthors and the elimination of one of the selected databases (i.e., Medline) to avoid duplication of records. One of the eligibility criteria was also modified to ensure that the systematic review included only patients with comorbid chronic pain and clinically relevant psychological distress (i.e., anxiety and/or depression), as well as exclusively in CBT-based interventions. Due to the heterogeneity of the characteristics of the clinical trials included in the systematic review and the small number of data explored for each outcome, it was not possible to compute the planned meta-analysis and meta-regression explained in the protocol. Finally, a narrative synthesis was performed in this systematic review.

## PRISMA checklist

| **Section/topic** | **#** | **Checklist item** | **Reported?** |
| --- | --- | --- | --- |
| **TITLE PAGE** | | |  |
| Title | 1 | Identify the report as a systematic review, meta-analysis, or both. | Yes |
| Funding | 2 | Describe sources of funding for the systematic review and other support (e.g., supply of data); role of funders for the systematic review on your title page. | Yes |
| Bulleted statements | 3 | 'Database?' and ' what does this review add?'. | - |
| **ABSTRACT** | | |  |
| Structured summary | 4 | Provide a structured summary including, as applicable: background and objective; databases and data treatment; results, conclusion; systematic review registration number. | Yes |
| **INTRODUCTION** | | |  |
| Rationale | 5 | Describe the rationale for the review in the context of what is already known. | Yes |
| Objectives | 6 | Provide an explicit statement of questions being addressed with reference to participants, interventions, comparisons, outcomes, and study design (PICOS). | Yes |
| **METHODS** | | |  |
| Protocol and registration | 7 | Indicate if a review protocol exists, if and where it can be accessed (e.g., Web address), and, if available, provide registration information including registration number. | Yes |
| Eligibility criteria | 8 | Specify study characteristics (e.g., PICOS, length of follow-up) and report characteristics (e.g., years considered, language, publication status) used as criteria for eligibility, giving rationale. | Yes |
| Information sources | 9 | Describe all information sources (e.g., databases with dates of coverage, contact with study authors to identify additional studies) in the search and date last searched. | Yes |
| Search | 10 | Present full electronic search strategy for at least one database, including any limits used, such that it could be repeated. | Yes |
| Study selection | 11 | State the process for selecting studies (i.e., screening, eligibility, included in systematic review, and, if applicable, included in the meta-analysis). | Yes |
| Data collection process | 12 | Describe method of data extraction from reports (e.g., piloted forms, independently, in duplicate) and any processes for obtaining and confirming data from investigators. | Yes |
| Data items | 13 | List and define all variables for which data were sought (e.g., PICOS, funding sources) and any assumptions and simplifications made. | Yes |
| Risk of bias in individual studies | 14 | Describe methods used for assessing risk of bias of individual studies (including specification of whether this was done at the study or outcome level), and how this information is to be used in any data synthesis. | Yes |
| **Section/topic** | **#** | **Checklist item** | **Reported?** |
| Summary measures | 15 | State the principal summary measures (e.g., risk ratio, difference in means). | Yes |
| Synthesis of results | 16 | Describe the methods of handling data and combining results of studies, if done, including measures of consistency (e.g., I^2^) for each meta-analysis. | Yes |
| Risk of bias across studies | 17 | Specify any assessment of risk of bias that may affect the cumulative evidence (e.g., publication bias, selective reporting within studies). | Yes |
| Additional analyses | 18 | Describe methods of additional analyses (e.g., sensitivity or subgroup analyses, meta-regression), if done, indicating which were pre-specified. | Yes |
| **RESULTS** | | |  |
| Study selection | 19 | Give numbers of studies screened, assessed for eligibility, and included in the review, with reasons for exclusions at each stage, ideally with a flow diagram. | Yes |
| Study characteristics | 20 | For each study, present characteristics for which data were extracted (e.g., study size, PICOS, follow-up period) and provide the citations. | Yes |
| Risk of bias within studies | 22 | Present data on risk of bias of each study and, if available, any outcome level assessment (see item 12). | Yes |
| Results of individual studies | 23 | For all outcomes considered (benefits or harms), present, for each study: (a) simple summary data for each intervention group (b) effect estimates and confidence intervals, ideally with a forest plot. | Yes |
| Synthesis of results | 24 | Present results of each meta-analysis done, including confidence intervals and measures of consistency. | Yes |
| Risk of bias across studies | 25 | Present results of any assessment of risk of bias across studies (see Item 15). | Yes |
| Additional analysis | 26 | Give results of additional analyses, if done (e.g., sensitivity or subgroup analyses, meta-regression [see Item 16]). | Yes |
| **DISCUSSION** | | |  |
| Summary of evidence | 27 | Summarize the main findings including the strength of evidence for each main outcome; consider their relevance to key groups (e.g., healthcare providers, users, and policy makers). | Yes |
| Limitations | 28 | Discuss limitations at study and outcome level (e.g., risk of bias), and at review-level (e.g., incomplete retrieval of identified research, reporting bias). | Yes |
| Conclusions | 29 | Provide a general interpretation of the results in the context of other evidence, and implications for future research. | Yes |

From Moher, D., Liberati, A., Tetzlaff, J., Altman, D. G., & The PRISMA Group. (2009). Preferred Reporting Items for Systematic Reviews and Meta-Analyses: The PRISMA Statement. *PLoS Medicine, 6*(6), e1000097. <https://doi.org/10.1371/journal.pmed1000097>

# Supplementary Table 2

**Characteristics of excluded studies**

| **Author (year)** | **Reason for exclusion** |
| --- | --- |
| Peniston et al. (1986) | No cognitive behavioral therapy-based intervention |
| Beutler et al. (1988) | No cognitive behavioral therapy-based intervention |
| Kemp et al. (2004) | Combination of pharmacological and CBT-based interventions |
| Zautra et al. (2008) | The entire sample did not have chronic pain and psychological distress |
| Ang et al. (2010) | Combination of pharmacological and CBT-based interventions |
| Wang et al. (2010) | No control group |
| Nash et al. (2013) | No psychological distress population |
| Scheidt et al. (2013) | No cognitive behavioral therapy-based intervention |
| Van Beek et al. (2013) | No chronic pain population |
| Poleshuck et al. (2014) | No cognitive behavioral therapy-based intervention |
| Hampel & Tlach (2015) | No psychological distress population |
| Karp et al. (2018) | Combination of pharmacological and CBT-based interventions |
| Aragonès et al. (2020) | Only cost-effectiveness analyses are presented |
| Sud et al. (2020) | No cognitive behavioral therapy-based intervention |

**References**

Ang, D. C., Bair, M. J., Damush, T. M., Wu, J., Tu, W., & Kroenke, K. (2010). Predictors of pain outcomes in patients with chronic musculoskeletal pain co-morbid with depression: Results from a randomized controlled trial. *Pain Medicine, 11*(4), 482–491. <https://doi.org/10.1111/j.1526-4637.2009.00759.x>

Aragonès, E., Sánchez-Iriso, E., López-Cortacans, G., Tomé-Pires, C., Rambla, C., & Sánchez-Rodríguez, E. (2020). Cost-effectiveness of a collaborative care program for managing major depression and chronic musculoskeletal pain in primary care: Economic evaluation alongside a randomized controlled trial.  *Journal of psychosomatic research*, *135*, 110167. https://doi.org/10.1016/j.jpsychores.2020.110167

Beutler, L. E., Daldrup, R., Engle, D., Guest, P., Corbishley, A., & Meredith, K. E. (1988). Family dynamics and emotional expression among patients with chronic pain and depression. *Pain*, *32*(1), 65–72. <https://doi.org/10.1016/0304-3959(88)90024-3>

Hampel, P., & Tlach, L. (2015). Cognitive-behavioral management training of depressive symptoms among inpatient orthopedic patients with chronic low back pain and depressive symptoms: A 2-year longitudinal study. *Journal of Back and Musculoskeletal Rehabilitation*, *28*(1), 49–60. <https://doi.org/10.3233/BMR-140489>

Karp, J. F., Gao, X., Wahed, A. S., Morse, J. Q., Rollman, B. L., Weiner, D. K., & Reynolds, C. F., 3rd (2018). Effect of Problem-Solving Therapy Versus Supportive Management in Older Adults with Low Back Pain and Depression While on Antidepressant Pharmacotherapy. The American journal of geriatric psychiatry. *Official Journal of the American Association for Geriatric Psychiatry, 26*(7), 765–777. <https://doi.org/10.1016/j.jagp.2018.01.004>

Kemp, B. J., Kahan, J. S., Krause, J. S., Adkins, R. H., & Nava, G. (2004). Treatment of major depression in individuals with spinal cord injury. *The Journal of Spinal Cord Medicine*, *27*(1), 22–28. <https://doi.org/10.1080/10790268.2004.11753726>

Nash, V. R., Ponto, J., Townsend, C., Nelson, P., & Bretz, M. N. (2013). Cognitive behavioral therapy, self-efficacy, and depression in persons with chronic pain. *Pain Management Nursing: Official Journal of the American Society of Pain Management Nurses*, *14*(4), e236–e243. <https://doi.org/10.1016/j.pmn.2012.02.006>

Peniston, E. G., Hughes, R. B., & Kulkosky, P. J. (1986). EMG biofeedback-assisted relaxation training in the treatment of reactive depression in chronic pain patients. The Psychological Record, 36(4), 471–481.

Poleshuck, E. L., Gamble, S. A., Bellenger, K., Lu, N., Tu, X., Sörensen, S., Giles, D. E., & Talbot, N. L. (2014). Randomized controlled trial of interpersonal psychotherapy versus enhanced treatment as usual for women with co-occurring depression and pelvic pain. *Journal of Psychosomatic Research, 77*(4), 264–272. <https://doi.org/10.1016/j.jpsychores.2014.07.016>

Sud, A., Nelson, M. L., Cheng, D. K., Armas, A., Foat, K., Greiver, M., Hosseiny, F., Katz, J., Moineddin, R., Mulsant, B. H., Newman, R. I., Rivlin, L., Vasudev, A., & Upshur, R. (2020). Sahaj samadhi meditation versus a health enhancement program for depression in chronic pain: Protocol for a randomised controlled trial and implementation evaluation. *Trials, 21*, 1-15. <https://doi.org/10.1186/s13063-020-04243-z>

Scheidt, C. E., Waller, E., Endorf, K., Schmidt, S., König, R., Zeeck, A., Joos, A., & Lacour, M. (2013). Is brief psychodynamic psychotherapy in primary fibromyalgia syndrome with concurrent depression an effective treatment? A randomized controlled trial. *General Hospital Psychiatry, 35*(2), 160–167. <https://doi.org/10.1016/j.genhosppsych.2012.10.013>

Zautra, A. J., Davis, M. C., Reich, J. W., Nicassario, P., Tennen, H., Finan, P., Kratz, A., Parrish, B., & Irwin, M. R. (2008). Comparison of cognitive behavioral and mindfulness meditation interventions on adaptation to rheumatoid arthritis for patients with and without history of recurrent depression. *Journal of Consulting and Clinical Psychology*, *76*(3), 408–421. <https://doi.org/10.1037/0022-006X.76.3.408>

Van Beek, M. H. C. T., Oude Voshaar, R. C., Beek, A. M., Van Zijderveld, G. A., Visser, S., Speckens, A. E. M., ... & Van Balkom, A. J. L. M. (2013). A brief cognitive‐behavioral intervention for treating depression and panic disorder in patients with noncardiac chest pain: A 24‐week randomized controlled trial. *Depression and Anxiety, 30*(7), 670-678. <https://doi.org/10.1002/da.22106>

Wang, H., Ahrens, C., Rief, W., & Schiltenwolf, M. (2010). Influence of comorbidity with depression on interdisciplinary therapy: outcomes in patients with chronic low back pain. *Arthritis Research & Therapy*, *12*(5), R185. <https://doi.org/10.1186/ar3155>

# Supplementary Table 3

**TABLE 3** Results of the controlled trials included in this systematic review.

| **Author (year)** | **Results** |
| --- | --- |
| **Cognitive behavioral therapy (CBT)** | |
| [1] Tlach et al. (2011) | Significant differences were identified in the decrease of depressive symptoms at post-treatment (*p* < 0.001, *d* = 1.31), follow-up+6 (*p* < 0.001, *d* = 0.89), follow-up+12 (*p* < 0.001, *d* = 0.75), and follow-up+24 (*p* = 0.01, *d* = 0.51), and anxiety symptoms at post-treatment (*p* < 0.001, *d* = 1.08), follow-up+6 (*p* < 0.001, *d* = 0.76), follow-up+12 (*p* = 0.001, *d* = 0.51) and follow-up+24 (*p* = 0.01, *d* = 0.43), as well as the increase of mental quality of life at post-treatment (*p* < 0.001, *d* = 0.78) and follow-up+6 (*p* < 0.01, *d* = 0.78), but not at the follow-up+12 and at follow-up+24, in CBT+TAU compared to TAU. |
| [2] Buhrman et al. (2015) | Significant differences were identified in the decrease of depressive symptoms (*p* = 0.004*, d* = 0.59), anxiety symptoms (*p* = 0.03, *d* = 0.34), and pain interference (*p* = 0.03, *d* = 0.12), as well as the increase of pain acceptance (*p* = 0.039, *d* = 0.12) at post-treatment, but not at the follow-up+12, in CBT+TAU compared to TAU. No significant differences between groups were found in the decrease of fear of anxiety symptoms, pain catastrophizing, cognitive and behavioral coping strategies, and psychosocial and behavioral consequence of chronic pain, as well as in the increase of quality of life at any assessment time. |
| [3] Migliorini et al. (2016) | Significant differences were identified in the decrease of depressive symptoms (*p* = 0.009*, d = 0.18*), anxiety symptoms (*p* = 0.001, *d = 0.52*), and stress symptoms (*p* = 0.008, *d = 0.47*), as well as the increase of quality of life (*p* = 0.02, *d = 0.58*) at post-treatment in favor of the CBT compared to waitlist. |
| [4] Ólason et al. (2017) | Significant differences were identified in the decrease of depression (*p* <.001, *d* = 0.57), anxiety (*p* <.001, *d* = 0.27) and in the improvement of social functioning (*p* = .004, *d* = 0.51) at follow-up+36, but not at the post-treatment and at follow-up+12, in favor of the CBT+TAU compared to TAU. No significant differences between groups were found in the decrease of pain intensity and fear avoidance at any assessment time. |
| [5] Aragonès et al. (2019) | Significant differences were identified in the decrease of depressive symptoms at follow-up+12 (*p* = 0.02, *d* = 0.26), but not at post-treatment and at follow-up+6, in CBT+TAU compared to TAU. No significant differences between groups were found in the decrease of pain intensity and pain interference at any assessment time. |
| [6] Boersma et al. (2019) | Significant differences between conditions were observed on pain interference (*p* < .05, *d* = 0.02) and pain catastrophizing (*p* < .05, *d* = 0.26) at post-treatment, but not at follow-up+9, as well as on pain interference (*p* = .02, *d* = 0.25) at follow-up+9 and depression symptoms (*p* = .04, *d* = 0.25) at follow-up+9, but not at post-treatment, in favor of the hybrid therapy (exposure in vivo and dialectical behavior therapy) compared to CBT. No significant differences between groups were found in the decrease of anxiety symptoms and pain intensity at any assessment time. |
| [7] Schlicker et al. (2020) | Significant differences were identified in the decrease of depressive symptoms at post-treatment (*p* = 0.01, *d* = 0.86), but not at follow-up+6, and anxiety symptoms at post-treatment (*p* < 0.001, *d* = 0.81) and follow-up+6 (*p* = 0.04, *d* = 1.07) in CBT+TAU compared to TAU. No significant differences between groups were found in the decrease of social functioning and pain intensity, as well as in the increase of quality of life, pain self-efficacy, and working capacity at any assessment time. |
| [8] Baumeister et al. (2020) | Significant differences were identified in the reduction of depressive symptoms at post-treatment (*p* = 0.038, *d* = 0.48) and at follow-up+6 (*p* = 0.047, *d* = 0.27) in CBT compared to TAU. A significant decrease in pain intensity and pain related disability was reported by patients assigned to CBT at post-treatment (*p* = 0.013, *d* = 0.42, *p* < 0.001, *d* = 0.35, respectively), but not at follow-up, compared to TAU. Significant improvements of pain self-efficacy and quality of life were detected at post-treatment (*p* < 0.001, *d* = 0.39, *p* < 0.001, *d* = 0.45, respectively) and at follow-up (*p* = 0.020, *d* = 0.33, *p* = 0.006, *d* = 0.35, respectively) compared to TAU. No significant differences between groups were found in the increase of working capacity at any assessment time. |
| [9] Gasslander et al. (2022) | Significant differences were identified in the decrease of depressive symptoms (*p* < 0.001, *d* = 0.18), anxiety symptoms (*p* < 0.01, *d* = 0.19), pain interference (*p* < 0.01, *d* = 0.22), and coping strategy of catastrophizing (*p* < 0.01, *d* = 0.38), as well as an in the increase of life control (*p* < 0.05, *d* = 0.10), pain acceptance (*p* < 0.01, *d* = 0.30), coping strategy of ignoring (*p* < 0.05, *d* = 0.34), and quality of life (*p* < 0.01, *d* = 0.02) at post-treatment in CBT compared to TAU. No significant differences between groups were found in the decrease of pain intensity, pain catastrophizing, fear of anxiety symptoms, and kinesiophobia, as well as in the increase of social functioning and pain self-efficacy. |
| **Mindfulness-based interventions (MBI)** | |
| [10] De Jong et al. (2016, 2018) | Significant differences were identified in the decrease of depressive symptoms (*p* = 0.04, *d* = 0.13) and the increase of quality of life (*p* = 0.01, *d* = 0.19), self-regulation (*p* < 0.01, *d* = .091) and emotional awareness (*p* = 0.04, *d* = 0.57) at post-treatment in MBI+TAU compared to TAU. No significant differences between groups were found in the decrease of anxiety symptoms, pain intensity, pain catastrophizing, and pain interference at post-treatment. |
| [11] Gardiner et al. (2019) | Significant differences were identified in the decrease of the physical composite (*p* < 0.005, *RR* = 0.86) and in the increase of the mental composite (*p* < 0.005, *RR* = 1.07) subscales of health-related quality of life at the follow-up+6, but not at post-treatment, in IMAGV+TAU compared to TAU. No significant differences between groups were found in the decrease of pain intensity and in the increase of pain self-efficacy, quality of life, and behavioral activation at any assessment time. |
| [12] Torrijos et al. (2021) | Significant differences between conditions were observed on self-compassion (*p* < 0.05, *d* = 0.05), pain interference (*p* < 0.05, *d* = 0.07), pain acceptance (*p* < 0.01, *d* = 0.19), pain catastrophizing (*p* < 0.10, *d* = 0.12), and symptoms of anxiety (*p* < 0.05, *d* = 0.17) at post-treatment in favor of the MSC compared to CBT. No significant differences between groups were found in the decrease of depression symptoms, as well as in the increase of quality of life at post-treatment. |
| **Acceptance and Commitment Therapy (ACT)** | |
| [13] Sanabria-Mazo et al. (2023) | Significant differences were found in the reduction of pain interference at post-treatment (*p* = 0.001, *d* = 0.64) and at follow-up+12 (*p* < 0.001, *d* = 0.73) in ACT+TAU compared to TAU. No significant differences between ACT+TAU and TAU were found in pain intensity, depressive, and anxiety symptoms. Stress was significantly reduced at post-treatment by ACT in comparison to TAU (*p* = 0.001, *d* = 0.69), but not at follow-up+12. A significant decrease in pain catastrophizing was reported by patients assigned to ACT+TAU at post-treatment (*p* = 0.001, *d* = 0.45) and at follow-up+12 (*p* = 0.002, *d* = 0.59) compared to TAU. Significant differences were found in the improvement of pain acceptance and psychological flexibility at post-treatment (*p* = 0.003, *d* = 0.34 and *p* < 0.001, *d* = 0.52, respectively) and at follow-up+12 (*p* = 0.002, *d* = 0.42 and *p* = 0.009, *d* = 0.37, respectively) in ACT+TAU compared to TAU. Finally, behavioral activation was significantly increased at post treatment by ACT in comparison to TAU (*p* = 0.024, *d* = 0.30), but not at follow-up+12.  No significant differences between ACT+TAU and BATD+TAU were found in pain interference, pain intensity, depressive, anxiety, and stress symptoms, pain catastrophizing, pain acceptance, behavioral activation, and psychological flexibility. |
| **Behavioral Activation Therapy for Depression (BATD)** | |
| [13] Sanabria-Mazo et al. (2023) | Significant differences were found in the decrease of pain interference at follow-up+12 (*p* = 0.001, *d* = 0.66) in BATD+TAU compared to TAU, but not at post-treatment. No significant differences between BATD+TAU and TAU were found in pain intensity, depressive, anxiety, and stress symptoms. A significant reduction in pain catastrophizing was reported by patients assigned to BATD+TAU at post-treatment (*p* = 0.001, *d* = 0.59) and at follow-up+12 (*p* = 0.002, *d* = 0.59) compared to TAU. Significant differences were found in the improvement of behavioral activation and psychological flexibility at post-treatment (*p* = 0.021, *d* = 0.46 and *d* = 0.40, respectively) in BATD+TAU compared to TAU, but not at the follow-up+12. Finally, no significant differences between BATD+TAU and TAU were found in pain acceptance.  No significant differences between ACT+TAU and BATD+TAU were found in pain interference, pain intensity, depressive, anxiety, and stress symptoms, pain catastrophizing, pain acceptance, behavioral activation, and psychological flexibility. |

# Supplementary Table 4

Upcoming controlled trial

| **Author (year), location, design** | **Target condition** | **Treatment arms (*n*); delivery period (format)** | **Sessions, *n* (minutes)** | **Therapists** | **Assessments (time horizon)** | **Primary outcome (instrument)** | **Secondary outcomes (instruments)** |
| --- | --- | --- | --- | --- | --- | --- | --- |
|  |  |  |  |  |  |  |  |
| Bell et al. (2020), Canada, RCT | CNCP and MDD | ACT, CBT, and TAU (online) | 7 (NR) | Graduate psychologist with the training and supervision of a senior psychologist | Pre, during, and post | - Depression symptoms (PHQ-9) | - Pain intensity (BPI) - Depression, anxiety, and stress symptoms (DASS-21) - Cognitive and behavioral coping strategies (CSQ-2IV) - Mental and physical well-being (SF-12) - Insomnia (ISI) - Perception of improvement after the intervention (PGIC) - Pain catastrophizing (PCS) - Social support (MSPSS) - Psychological flexibility (MPFI) |
| *Note*. ACT = acceptance and commitment therapy; BPI = Brief Pain Inventory; CBT = cognitive behavior therapy; CNCP = chronic non-cancer pain; CSQ-2IV56 = Coping Strategies Questionnaire-2-Item Version; RCT = randomized controlled trial; DASS-21 = Depression Anxiety Stress Scale; ISI = Insomnia Severity Index; MDD = major depressive disorder; MPFI = Multidimensional Psychological Flexibility Inventory; MSPSS = Multidimensional Scale of Perceived Social Support; NR = Not Reported; PCS = Pain Catastrophizing Scale; PGIC = Patient Global Impression of Change; PHQ-9 = Patient Health Questionnaire; SF-12 = 12-Item Short-Form Health Survey; TAU = treatment-as-usual. | | | | | | | |
